# On the Role of Curved Membrane Nanodomains and Passive and Active Skeleton Forces in the Determination of Cell Shape and Membrane Budding

**DOI:** 10.3390/ijms22052348

**Published:** 2021-02-26

**Authors:** Luka Mesarec, Mitja Drab, Samo Penič, Veronika Kralj-Iglič, Aleš Iglič

**Affiliations:** 1 Faculty of Electrical Engineering, University of Ljubljana, SI-1000 Ljubljana, Slovenia; luka.mesarec@fe.uni-lj.si (L.M.); mitja.drab@fe.uni-lj.si (M.D.); samo.penic@fe.uni-lj.si (S.P.); 2 Faculty of Health Sciences, University of Ljubljana, SI-1000 Ljubljana, Slovenia; veronika.kralj-iglic@zf.uni-lj.si; 3 Institute of Biosciences and Bioresources, National Research Council, 80131 Napoli, Italy

**Keywords:** cytoskeleton, membrane skeleton, cell shape, orientational ordering, actin filaments, active force, BAR domains, anisotropic shape of molecules, NMIIA motor domains, membrane budding

## Abstract

Biological membranes are composed of isotropic and anisotropic curved nanodomains. Anisotropic membrane components, such as Bin/Amphiphysin/Rvs (BAR) superfamily protein domains, could trigger/facilitate the growth of membrane tubular protrusions, while isotropic curved nanodomains may induce undulated (necklace-like) membrane protrusions. We review the role of isotropic and anisotropic membrane nanodomains in stability of tubular and undulated membrane structures generated or stabilized by cyto- or membrane-skeleton. We also describe the theory of spontaneous self-assembly of isotropic curved membrane nanodomains and derive the critical concentration above which the spontaneous necklace-like membrane protrusion growth is favorable. We show that the actin cytoskeleton growth inside the vesicle or cell can change its equilibrium shape, induce higher degree of segregation of membrane nanodomains or even alter the average orientation angle of anisotropic nanodomains such as BAR domains. These effects may indicate whether the actin cytoskeleton role is only to stabilize membrane protrusions or to generate them by stretching the vesicle membrane. Furthermore, we demonstrate that by taking into account the in-plane orientational ordering of anisotropic membrane nanodomains, direct interactions between them and the extrinsic (deviatoric) curvature elasticity, it is possible to explain the experimentally observed stability of oblate (discocyte) shapes of red blood cells in a broad interval of cell reduced volume. Finally, we present results of numerical calculations and Monte-Carlo simulations which indicate that the active forces of membrane skeleton and cytoskeleton applied to plasma membrane may considerably influence cell shape and membrane budding.

## 1. Introduction

**Figure 1:**
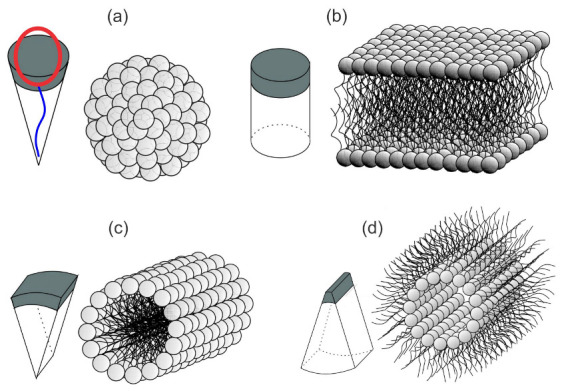
Schematically shown polymorphisms of phospholipid aggregates with the corresponding (isotropic and anisotropic) shapes of phospholipid molecules. Panel (**a**) shows a spherical micelle and a conically shaped lipid with a hydrophilic head (red) and a hydrophobic tail (blue). Figure also shows a phospholipid bilayer (**b**), a cylindrical micelle (**c**) and an inverted cylinder (**d**). Adapted with permission from ref. [5]. 2009 Elsevier.

The lipid bilayer, embedded with inclusions like proteins and lipids, is the main element of biological membranes [1,2]. A typical biological membrane contains three types of lipid molecules-phospholipids, glycolipids and cholesterol [3], where the majority of the lipid bilayer is composed of phospholipids [4]. A lipid molecule consists of hydrophilic (polar) head and hydrophobic (non-polar) tail composed of fatty chains [4]. The shape and curvature of pure lipid structures depend on the intrinsic shape of phospholipid molecules (see Figure 1). For example, if the lipids are conically shaped, they can aggregate densely and form a micelle (Figure 1a), which is the smallest object formed by aggregation of single-chained lipids (surfactants). In this case, the hydrophobic tails are oriented towards each other, away from the water solution, while the hydrophilic heads are in contact with water [4] (Figure 1a). Futhermore, if the lipids are “cylindrically” shaped, they tend to self-assemble into planar lipid bilayers (Figure 1b) [2,4]. Only “cylindrically” shaped phospholipid molecules tend to self-assemble into planar bilayer structures (Figure 1b). Phospholipid molecules which are not “cylindrically” shaped self-assemble into non-planar structures (see panels (a,c,d) in Figure 1). In general, phospholipid molecules with two tails are anisotropic (including “cylindrically” shaped phospholipids), which can result in formation of anisotropic phospholipid aggregates (see panels (c,d) in Figure 1). Note that at the edge of the lipid bilayer, hydrophobic tails would be still in contact with water (Figure 1b), which can be avoided by the formation of closed membrane shapes-vesicles [4].

**Figure 2:**
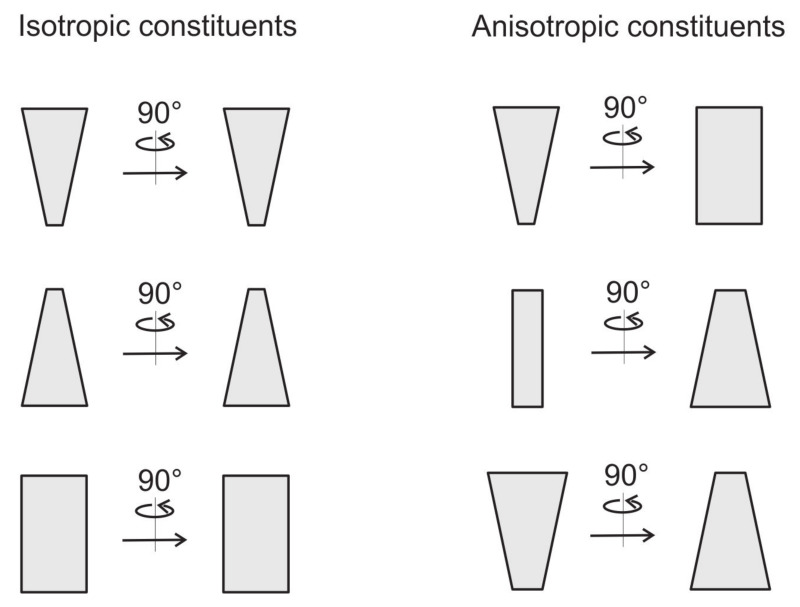
Schematic representation of different isotropic ($C_{1m}=C_{2m}$) and anisotropic ($C_{1m}\neq C_{2m}$) shapes of lipid bilayer-embedded constituents (inclusions). Front and side views of inclusions are shown.

Biological membrane takes part in different biological processes, such as the trans-membrane transport of nutrients, envelopment of larger particles or viruses, communication between cells and cell’s waste control [6,7]. Biological membranes can be viewed as a complex multicomponent system [2,8], composed of lipid molecules, proteins, carbohydrates and many other biologically active components [9]. These components/inclusions may promote local membrane curvature changes, sometimes resulting in a global adjustment of the cell shape [10,11,12,13,14,15,16,17,18,19,20,21]. Membrane shape depends on the intrinsic shape of membrane constituents and their interactions with other constituents, membrane skeleton and cytoskeleton. It has been shown that a non-homogeneous lateral distribution and phase separation of membrane inclusions may be a driving force of cell shape transformations [13,14,15,16,19,22,23,24,25,26,27,28,29]. Different proteins, lipid molecules and their complexes are able to move freely within some region of the two-dimensional membrane [23,30]. The lipid bilayer contains various kinds of proteins and other molecules which have anisotropic properties (see Figure 2) [31,32,33,34,35,36]. Also, lipid molecules, the main component of biological membranes, are in general anisotropic (see Figure 1) [37,38,39].

Besides by membrane inclusions, the shapes of cells and/or lipid bilayer vesicles (often used as model systems) may be influenced also by the cytoskeleton and membrane skeleton and their forces [20,27,40,41,42,43,44,45,46]. Among these are ATP -consuming active forces important for different cell functions [19,24,47,48,49,50,51]. Consequently, new theoretical approaches for modelling changes in the cell shape as a consequence of energy-consuming active forces have recently been developed [19,24,47,50,51].

For example, in the case of the red blood cells (RBC) it was long believed that active forces are totally absent in the mechanisms of cell shape determination; a belief that was refuted only recently [48,49,50]. It has been shown that myosin (NMIIA) motor nanodomains may cause tension in the spectrin-F-actin RBC membrane skeleton and therefore partially control the RBC shape [48,49] and membrane vesiculation [50].

The non-homogeneous distribution of NMIIA motor nanodomains over the whole inner surface of discocyte RBC membrane is in accordance with experimental observations [49]. It was suggested that in order to keep a stable discocyte shape of RBC and prevent a pancake shape transformation, the normal component of the NMIIA nanodomain force should be directed to the interior of the cell and different than zero [49]. This applies to the whole surface of the membrane, including the dimple region of the dicocyte RBC shape [49]. If the NMIIA motor proteins are contracted in the dimple region of the RBC, they may induce modest local exvaginations and non-zero components of myosin force directed into the RBC interior. Since actin molecules and NMIIA motor nanodomains are distributed only on the inner surface of the RBC membrane, the normal component of the NMIIA motor nanodomain is always directed inward.

Experimental measurements of NMIIA densities of RBC discocytes at the rim and dimple confirmed the theoretical predictions [49] that the NMIIA force density has to be larger in the dimple compared to the RBC rim in order to stabilize the discocyte RBC shape [49]. It was also shown that a decrease in the difference between the outer and inner lipid layer relaxed areas induced the inward bending of the RBC membrane [46,52,53,54,55], while an increase between the relaxed areas of the outer and inner membrane layers favored outward bending [45,46,52,53,54,55,56]. Consequently, exogenously added amphiphiles which bind predominantly in the outer lipid layer induce the transformation of a discoid RBC into a spiculated RBC shape (echinocytic), while amphiphiles bound predominantly in the inner lipid layer promote the transformation into invaginated stomatocytic shapes [54,55,57].

In addition to lipid bilayer, membrane of RBC contains also a membrane skeleton, which is composed of a spectrin-F-actin network attached to the inner surface of the lipid bilayer [43]. This implies that a shear elastic energy of the membrane skeleton should also be included alongside the local and non-local bending energies in the free energy minimization of the membrane in order to explain the observed stability of spiculated (echinocytic) RBC shapes theoretically [42,44,45,46,47,52,58,59,60,61,62,63,64,65,66]. Recently, it was also shown that in RBCs, the ATP-dependent membrane skeleton forces, which are exerted on the membrane by the skeleton nodes, may cause also membrane softening, which may possibly influence the RBC deformability to facilitate the migration of RBCs through narrow capillaries [47].

In cellular processes, tubular protrusions of biological membranes have an important role. Membrane nanotubes that are formed by lipid bilayers are universal in cell biology [29,67,68,69,70,71]. The stability and growth of membrane tubular structures, observed in experiments, can be explained theoretically. It was shown that minimisation of the isotropic bending energy of the membrane introduced by Helfrich [72,73] is not sufficient to predict the stability of membrane tubular protrusions [23,26,74,75].

Early physical models [72,73] considered the biological membrane as a thin elastic shell with isotropic properties. These models along with their modifications successfully described some of the experimentally observed shapes of erythrocytes and phospholipid vesicles in cases where the membrane does not exhibit regions of high anisotropic curvature (reviewed in [76]). In order to theoretically study also membrane shapes with highly curved anisotropic regions, a model considering deviatoric elasticity was proposed within a continuum approach [77,78], introducing the spontaneous membrane warp as a parameter. However, it was assumed that its value is negligible [77,78], also because the existence of the membrane nanostructures was at that time not yet widely acknowledged and biological membranes were considered locally nearly flat. In 1996, the deviatoric elasticity (DE) model was proposed, which takes into account also the anisotropic properties of membrane components [79,80]. Deviatoric membrane free energy was derived from an energy of a single-constituent applying the methods of statistical physics [15,23,74,79,80,81]. In general, membrane constituents/nanodomains in the DE model can be considered isotropic or anisotropic [8,15,25,75,80,82,83,84]. DE model can theoretically explain stable shapes of cells and vesicles possessing strongly anisotropically curved regions, for example shapes with thin tubular protrusions [21,23,75,85,86,87,88,89,90] and narrow necks [15,16,26,81,91].

**Figure 3:**
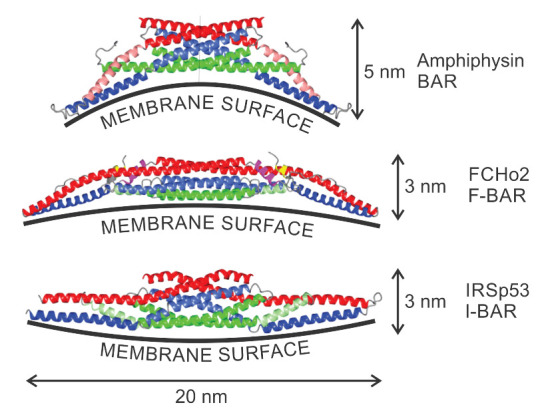
Schematic presentation of the Bin/Amphiphysin/Rvs (BAR) superfamily domains. BAR domains are shown along with their typical sizes and curvature preferences. Adapted with permission from refs. [18,108]. 2016 Elsevier.

The aggregation of different proteins in biological membranes is important for normal cell functioning. Furthermore, a disruption in key mechanisms of protein aggregation in membranes may lead to neurodegenerative diseases [18,92]. Local accumulation of anisotropic membrane proteins may induce the cell membrane tubulation [71,93,94]. The tubulation of the cell membrane could be spontaneous or induced by external agents [26,67,70,74,75,89,93,95,96,97,98,99,100,101,102]. Typical example of anisotropic proteins are for example Bin/Amphiphysin/Rvs (BAR) superfamily protein domains [103] (see Figure 3). These can change the local and global membrane curvature resulting in formation of the membrane tubular structures [103]. In epithelial and endothelial cell tissues, held together by cell-cell junctions containing transmembrane and cytoplasmic proteins, unbalances in cytoskeletal derived forces can cause the bending of the junctional membrane, which in turn triggers the recruitment of curved BAR proteins to the positively curved junctional interface [104,105,106,107].

BAR superfamily domains are named after the three proteins in which they are found, namely Bin, Amphiphysin and Rvs. These domains are able to induce the local bending of the membrane if the binding energy of the domain is larger than the energy required to bend the membrane [103]. BAR superfamily domains can dimerise and they may be involved in membrane association [33,109]. Furthermore, members of the BAR superfamily are important for cell migration, cell division, membrane trafficking and organelle biogenesis [18,110]. The interaction between different proteins and membranes may result in shape deformations and changes in topology of cell/vesicle membranes. This effect is called protein mediated membrane remodeling [111,112]. BAR protein domain induced membrane remodeling can result in tubulation and vesiculation of liposomes [18,113].

The BAR domain superfamily contains BAR/N-BAR, F-BAR and I-BAR domains, each with a different local curvature preference, i.e., the membrane binding surface areas of the BAR/F-BAR and the IMD/I-BAR domains have opposite curvatures [18,114,115] (see Figure 3). The BAR protein domain is a bow-shaped dimer that binds preferentially to negatively charged membranes with high positive curvature [18,33,116,117]. The amphiphysin BAR dimer is capable of stabilizing the membrane curvature [118] and tubulating liposomes in vitro [119]. The F-BAR modules also have a preference to bind to membrane regions with a positive curvature, but they have lower intrinsic curvature than the BAR/N-BAR modules (see Figure 3). F-BAR domains could also tubulate membranes [18]. The overexpression of the pacsin2 EFC/F-BAR may induce cellular microspikes and deform liposomes into tubules in vitro [120]. I-BAR modules on the other hand, preferentially bind to membrane surfaces with negative curvature [18,121] (see Figure 3). I-BAR domains are also capable of deforming biological membranes and inducing the growth of plasma membrane protrusions, while attaching to the inner side of the membrane of tubular protrusion [71,93]. The membrane deforming activity of I-BAR domains of IRSp53 proteins is crucial for the bending of membrane patches into filopodia protrusions [122,123]. It was shown that IMDs can induce the development of membrane tubular invaginations protruding into the liposome interior [122]. The I-BAR IRSp53 domains, attached to the inner membrane surfaces, could induce dynamic membrane protrusions without actin that are thinner than normal filopodia [18,100].

Membrane tubular protrusions may also form as a consequence of force acting on the membrane. External force, stretching the membrane, can be for example generated experimentally by the tip of the cantilever to which the force of the atomic force microscope is applied [41]. The surface of a cell membrane could also be deformed in a small region when it is subjected to a localized torque or force caused by an integral protein, a receptor or a cell-kicking instrument [41]. In experiments with liposomes, the origin of such protrusive forces can also stem from the polymerization of actin, which leads to the growth of prolonged actin filaments beneath the membrane [124,125,126]. The elongating actin filaments could “push out” the cell/vesicle membrane in direction normal to the surface [124], which may result in the formation of membrane tubes [23]. However, it is still not completely clear whether the membrane tubular protrusions are pushed and deformed by the polymerizing actin filaments inside the cell, or they are only stabilized by actin filaments [18,93]. It has been indicated that actin filaments are not decisive for the generation of a membrane protrusion, but only for stabilization of the initial protrusion induced by BAR proteins or pentasaccharide GM1 [71,93,110,127,128]. The transport of proteins to the tip of the protrusion via myosin motors is crucial for the formation and maintenance of cellular protrusions based on actin [129]. On the other hand, experimental and theoretical studies suggest that some of membrane tubular protrusions could be stable and even grow without actin filaments [23,26,74,75,87].

In general, the actin cytoskeleton represents a network of protein filaments stretching over the cytoplasm [30]. A lipid bilayer can cause the emergence of bundled actin filament protrusions from branched actin filament networks, which implies that the membrane actively participates in organizing actin filaments [130]. It was shown that the coupling between actin and curved proteins can induce membrane instabilities [125,131]. Curved proteins that mobilize actin can destabilize the membrane tube either as a result of the inward force forming denser protein rings that shrink the tube [131] or due to fast squeeze, leading to pearling of a uniform actin coat [125]. Furthermore, the pearling instability can initiate the membrane tube fission into distinct vesicles [23,125].

**Figure 4:**
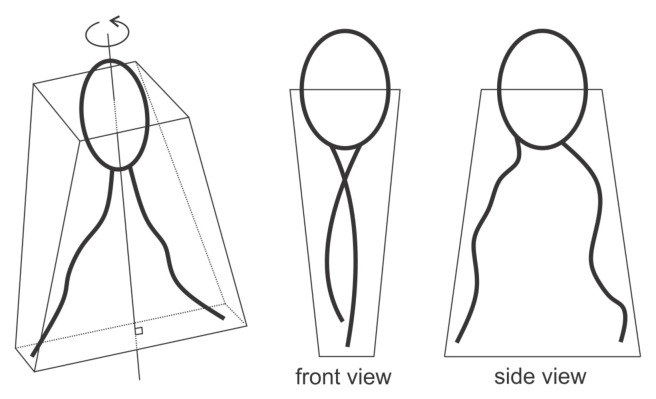
In-plane orientational order within the membranes of red blood cells or extracellular vesicles could arise due to V-shaped stretched chains of phospholipids. Adapted with permission from ref. [137]. 2019 Springer Nature.

Biological membranes very likely possess some degree of in-plane ordering, especially in membrane regions with high and anisotropic curvature [15,79,80]. For example, orientational nematic ordering may occur because of the membrane attached rod-like BAR domains in regions with high concentrations of BARs, where the rotation of an individual BAR domain becomes restricted due to direct/steric interaction with neighboring BAR domains [18,132]. Nematic ordering might occur also due to two flexible hydrocarbon chains of lipids [37,81,133] (Figure 4). Furthermore, tilt and hexatic orientational ordering may be developed by the tails of lipid molecules that tilt relative to the surface normal [82,134,135]. The orientational in-plane order in hexatic membranes with short-range positional order and long-range bond orientation order has been observed experimentally [136]. Within a statistical-mechanical approach, it has been indicated that in some membrane regions, the average orientation of lipid molecules cannot be neglected in spite of their rotational movement [81].

## 2. Modeling of Closed Membrane Shapes

### 2.1. Deviatoric Elasticity Model

**Figure 5:**
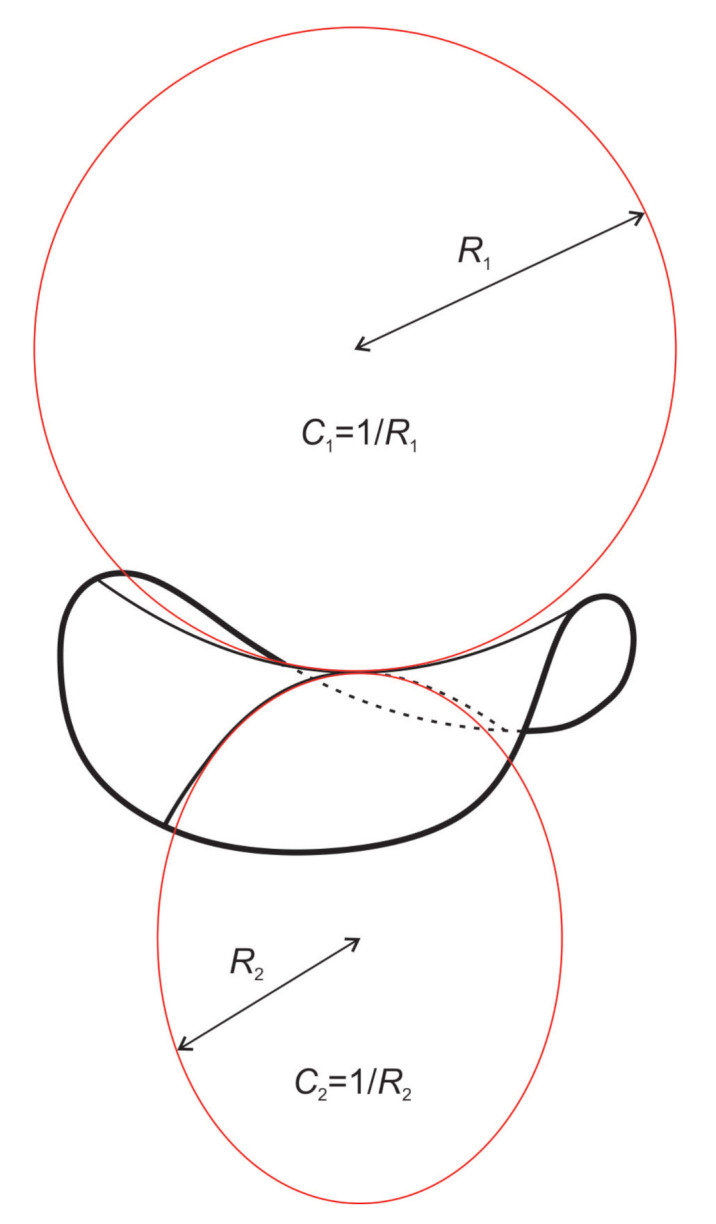
Schematic presentation of the two principal curvatures ($C_{1}$ and $C_{2}$) of a saddle-like surface. The first principal curvature is negative $\left(C_{1}<0\right)$, while the second one is positive $\left(C_{2}>0\right)$.

Local membrane shape is described by the two principal membrane curvatures $C_{1}$ and $C_{2}$ (see Figure 5), while the intrinsic shape of the membrane constituent (see Figure 2) is characterized by the corresponding principal curvatures $C_{1m}$ and $C_{2m}$ of the imaginary membrane into which the unconstrained constituent would fit perfectly [15,26,75,80]. Each membrane constituent may also locally rotate with respect to the membrane [15,84]. If the two principal curvatures of the membrane at a given location are equal ($C_{1}=C_{2}$), the local membrane shape is considered isotropic, while if they differ ($C_{1}\neq C_{2}$), it is considered anisotropic [26,74,79,80]. Likewise, if the intrinsic principal curvatures of membrane constituent are equal ($C_{1m}=C_{2m}$), the constituent is considered isotropic, while if they differ ($C_{1m}\neq C_{2m}$), it is considered anisotropic [15,18,26,79,80,83,84] (see also Figure 2).

**Figure 6:**
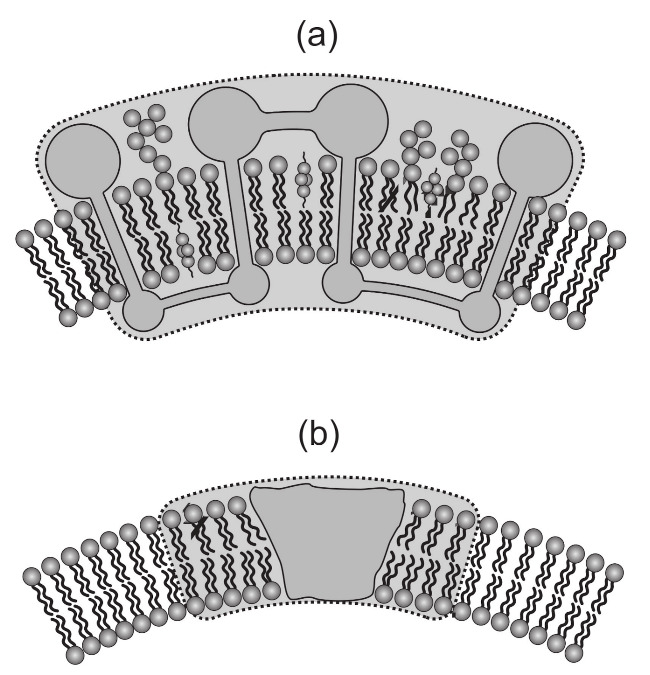
Schematic presentation of flexible membrane nanodomains (consisting of proteins and lipids), which are denoted by shaded area. Panel (**a**) shows a flexible membrane nanodomain including a flexible chain-like protein [26], while panel (**b**) shows a flexible membrane nanodomain formed by a membrane-embedded rigid protein [12,21,22].

The energy of a single membrane element (molecule or nanodomain, Figure 1, Figure 4 and Figure 6) is assumed to be a function of the mismatch between curvature tensors $\underline{C}$ and ${\underline{C}}_{m}$, which are defined as (Equations (1) and (2)):

(1) $$\underline{C}=\left[\begin{array}{c} C_{1}\;0\\ 0\;C_{2} \end{array}\right]\;,$$

(2) $${\underline{C}}_{m}=\left[\begin{array}{c} C_{1m}\;0\\ 0\;C_{2m} \end{array}\right]\;,$$

where $C_{1}$ and $C_{2}$ are the principal curvatures of the membrane surface (see Figure 5), and $C_{1m}$ and $C_{2m}$ are the intrinsic principal curvatures of the membrane element [21,90]. In this formalism, the membrane element can have anisotropic properties if $C_{1m}\neq C_{2m}$ (see Figure 1 and Figure 2). In general, the curvature tensors ${\underline{C}}_{m}$ and $\underline{C}$ have different orientations, i.e., they are rotated by an angle ω. The mismatch tensor $\underline{M}$ (Equation (3)) is introduced to express the mismatch between ${\underline{C}}_{m}$ and $\underline{C}$:

(3) $$\underline{M}=\underline{R}\;{\underline{C}}_{m}{\underline{R}}^{-1}-\underline{C}\;,$$

where the transformation matrix for rotation $\underline{R}$ (Equation (4)) is expressed as [21,90]:

(4) $$\underline{R}=\left[\begin{array}{c} \cos\omega \;-\sin\omega \\ \sin\omega \;\cos\omega \end{array}\right]\;.$$

Each membrane element should adapt so that it fits into its place in the membrane [90]. We will estimate the energy which is needed for the deformation of the membrane surface. The elastic energy density *w* should be a scalar quantity, which means that each term in the *w* expansion must also be a scalar. Therefore, *w* (Equation (5)) may be expressed by two invariants of the tensor $\underline{M}$, trace and determinant [84]:

(5) $$w=\frac{K_{1}}{2}{\left(\mathrm{Tr}\left(\underline{M}\right)\right)}^{2}+K_{2}\mathrm{Det}\left(\underline{M}\right)\;,$$

where $K_{1}$ and $K_{2}$ are constants [21,90]. Equation (5) can be rewritten as (Equation (6)):

(6) $$w=\left(2K_{1}+K_{2}\right){\left(H-H_{m}\right)}^{2}-K_{2}\left(D^{2}-2DD_{m}\cos\left(2\omega \right)+D_{m}^{2}\right)\;,$$

where $H=(C_{1}+C_{2})/2$ is the mean curvature of the membrane, $H_{m}=\left(C_{1m}+C_{2m}\right)/2$ is the mean intrinsic (spontaneous) curvature of the membrane element, $D=(C_{1}-C_{2})/2$ is the curvature deviator of the membrane, and $D_{m}=\left(C_{1m}-C_{2m}\right)/2$ is the intrinsic curvature deviator of the membrane element [15,21,74,77,78,79,80,83,90]. We can clearly see from Equation (6) that the elastic energy of anisotropic membrane element ($D_{m}\neq 0$) directly depends on its orientation ω. Therefore, the orientational ordering of anisotropic membrane constituents is also considered in this formalism [75,81].

### 2.2. Elastic Constants and Intrinsic Curvatures of Protein-Induced Nanodomain as a Function of Lipid and Protein Properties

In this section, we estimate the phenomenological parameters $H_{m}$, $D_{m}$, $K_{1}$ and $K_{2}$ (Equation (6)) of membrane nanodomain induced by anisotropic rigid protein (Figure 6b) using a simple theoretical model of the lipid bilayer elasticity [12,21]. We will assume that the local microscopic deformations of the membrane shape in the vicinity of the membrane-embedded rigid protein are constrained (Figure 6b) and the lipids adjust their tilt due to the intrinsic shape of the rigid protein [21].

**Figure 7:**
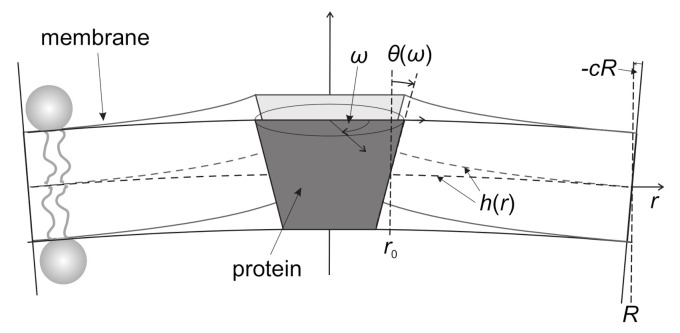
Schematic illustration of conical membrane-embedded rigid protein. For anisotropic proteins, the cone angle θ depends on the azimuthal angle ω. Adapted with permission from ref. [22]. 2006 APS.

Let us consider a single anisotropic cone-like rigid protein. To introduce anisotropy of the protein, we assume that the cone angle θ=θ(ω) depends on the azimuthal angle ω (Figure 7). For small θ variations we can write (Equation (7)) [21]

(7) $$\theta \left(\omega \right)=\bar{\theta }+\Delta \theta \cos\left(2\omega \right)\;,$$

where $\bar{\theta }$ represents the average conicalness of the protein and Δθ the difference between the maximal and minimal conicalness.

For the sake of simplicity, it is assumed that the rigid protein is embedded in one lipid layer only [12], with its local shape defined by mean curvature *H* and deviatoric curvature *D*. According to the Euler’s lemma, the line curvature of the normal cross section measured in the radial direction of the protein inclusion at azimuthal angle ω is given by Equation (8)

(8) C(ω)=H+Dcos(2ω).

The free energy perturbation of the lipid bilayer, induced by the rigid protein, can be expressed as an integral of the free energy density $\tilde{E}\left(\omega \right)$ per unit length of the circumference of the protein inclusion’s core. The circumference length is $L=2\pi r_{0}$, where $r_{0}$ is the radius of the inclusion’s core (Figure 7). The free energy is therefore expressed as: $E=\int_{L}\tilde{E}dL=\left(L/2\pi \right)\int \tilde{E}\left(\omega \right)d\omega$. For sufficiently large rigid protein inclusion radius $r_{0}$, we expect that $\tilde{E}=\tilde{E}\left[C\left(\omega \right),\theta \left(\omega \right)\right]$ is a function of ω via the relations C(ω) and θ(ω) [12]. In general, $\tilde{E}$ should depend also on the derivatives of C(ω) and θ(ω) with respect to ω. The dependence on the derivatives should become relevant if the radius of the inclusion $r_{0}$ is smaller than the characteristic decay length of the membrane perturbations ζ. Using membrane elasticity theory, ζ has been calculated [138] for a planar lipid layer that is in contact with a wall tilted by an angle θ. The value of ζ depends on the thickness of the lipid bilayer, the tilt modulus and the lateral stretching modulus. Typical values of ζ for a lipid monolayer [12,21] are about 1nm. Therefore, by assuming that $r_{0}\geq \zeta$, we can write (Equation (9))

(9) $$\frac{E}{L}=\frac{1}{2\pi }\int_{0}^{2\pi }\tilde{E}\left[C\left(\omega \right),\theta \left(\omega \right)\right]d\omega \;.$$

In above equation, $\tilde{E}$ can be calculated by using a one-dimensional model for the elastic interaction of a lipid layer with a rigid wall. Such a model from previous works [138,139] can be generalized in order to describe a bent lipid layer of curvature *C* (Equation (10)) [12,21]:

(10) $$\tilde{f}\left(C,\theta \right)=\frac{\kappa_{0}}{2\zeta }{\left(\theta -Cr_{0}\right)}^{2}+\left(C_{0}-C\right)\left(\theta -Cr_{0}\right)\;,$$

where $\kappa_{0}$ is the bending stiffness of the monolayer and $C_{0}$ its spontaneous curvature. We can substitute θ(ω) from Equation (7) and C(ω) from Equation (8) into Equation (10) and compare the obtained free energy density f(C,θ) with the elastic free energy density *w* from Equation (6), which yields (Equation (11)) [12,21]:

$$H_{m}=\frac{\bar{\theta }}{r_{0}}\left(\frac{r_{0}+\zeta }{r_{0}+2\;\zeta }\right)+\frac{\zeta C_{0}}{r_{0}+2\;\zeta }\;\;,\;D_{m}=\frac{\Delta \theta }{r_{0}}\left(\frac{r_{0}+\zeta }{r_{0}+2\;\zeta }\right)\;,$$

(11) $$K_{1}=\frac{3\pi }{4}r_{0}^{2}\kappa_{0}\left(\frac{r_{0}}{\zeta }+2\right)\;,\;\;K_{2}=-\frac{\pi }{2}r_{0}^{2}\kappa_{0}\left(\frac{r_{0}}{\zeta }+2\right)\;.$$

This demonstrates how the shape of the membrane-embedded rigid protein is included in the expressions for the rigid protein induced flexible nanodomain (Figure 6b) intrinsic mean curvature and the intrinsic curvature deviator: $H_{m}∼\bar{\theta }/r_{0}$ and $D_{m}∼\Delta \theta /r_{0}$. Strong dependence of the constants $K_{1}∼r_{0}^{3}$ and $K_{2}∼r_{0}^{3}$ on the protein radius (for $r_{0}\gg \zeta$) is a consequence of both the membrane-embedded protein rigidity (contributing $∼r_{0}^{2}$) and the linear increase of the circumference with $r_{0}$.

**Figure 8:**
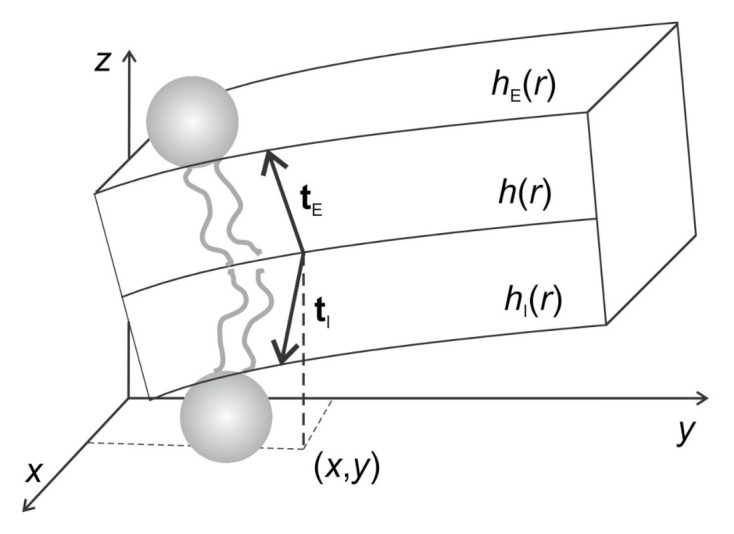
Schematic presentation of a perturbed lipid bilayer with local directors $t_{E}$ and $t_{I}$ and height profiles $h_{E}$ and $h_{I}$ of the external and internal membrane leaflet, respectively. The average bilayer height is $h=(h_{E}+h_{I})/2$. Adapted with permission from ref. [22]. 2006 APS.

Within more advanced model [21,22], the tilt deformation can be explicitly taken into account and the lipid membrane consisting of two opposed monolayers, an external one (E) and an internal one (I), can be also considered. The external and the internal monolayer were described by their height profiles ($h_{E}$ and $h_{I}$) and by their local directors ($t_{E}$ and $t_{I}$), which describe the average orientation of the lipid chains as shown in Figure 8 [21,22].

For the external monolayer, the elastic free energy per unit area (Equation (12)) can be written up to quadratic order in $h_{E}$ and $t_{E}$ as [21,22]:

(12) $$\begin{array}{ccc} {\hat{f}}_{E} & = & \frac{\kappa_{s}}{2}{\left(\Delta \cdot t_{E}\right)}^{2}+\frac{\kappa_{t}}{2}{\left(t_{E}-\Delta h_{E}\right)}^{2}+\frac{B}{2}{\left(h_{E}-h\right)}^{2}\\ & + & \frac{\kappa_{h}}{2}{\left(\Delta h_{E}\right)}^{2}+\frac{K}{2}{\left(\Delta \times t_{E}\right)}^{2}+\bar{\kappa }\det h_{E,ij}\;. \end{array}$$

The first term in Equation (12) describes the splay energy of the lipid chains, where $\kappa_{s}$ stands for the corresponding splay modulus. The second term takes into account the energy penalty of tilting the director $t_{E}$ relative to the normal of the surface *E*, where $\kappa_{t}$ is the tilt modulus [73]. It was shown that lipid tilt degree of freedom may lead to local softening of membrane regions with embedded rigid proteins (Figure 6b) [21,22]. The third term accounts for the thickness changes of the monolayer, where *B* represents the compression modulus and *h* a reference surface with respect to which the compression or expansion of the monolayer is measured. The bare bending energy of the external monolayer is expressed by the fourth term, where $\kappa_{h}$ is the corresponding modulus. Note that the splay energy (the first term) accounts mainly for the splay deformation of the lipid chains, while the bending energy (the fourth term) originates mostly in the monolayer headgroup region. Finally, the last two terms in Equation (12) take into account the twist deformation of the chains (modulus *K*) and the saddle deformation of $h_{E}$ (modulus $\bar{\kappa }$) [21,22].

The elastic free energy of the internal membrane leaflet ${\hat{f}}_{I}$ is obtained by replacing $h_{E}$ with $h_{I}$ and $t_{E}$ with $-t_{I}$ in the expression for ${\hat{f}}_{E}$ given by Equation (12). The minus sign is reflecting the opposite orientation of the opposed monolayers. The elastic energies from both monolayers contribute to the total free energy of the lipid bilayer: ${\hat{f}}_{bl}={\hat{f}}_{E}+{\hat{f}}_{I}$ [21,22].

### 2.3. Isotropic Limit and Helfrich Model

If the membrane contains only isotropic components (for example isotropic lipids or protein-induced nanodomains [22]) with the intrinsic (spontaneous) curvature $C_{1m}=C_{2m}$, i.e., $D_{m}=0$ in Equation (6), the elastic energy density becomes (Equation (13)):

(13) $$w=\left(2K_{1}+K_{2}\right){\left(H-H_{m}\right)}^{2}-K_{2}D^{2}=\frac{K_{1}}{2}{\left(2H-C_{0}\right)}^{2}+K_{2}K+K_{1}{C_{0}}^{2}\left(\frac{K_{1}}{2K_{1}+K_{2}}-\frac{1}{2}\right),$$

where $K=C_{1}C_{2}$ is the Gaussian curvature and (Equation (14)) [90]

(14) $$C_{0}=\frac{\left(2K_{1}+K_{2}\right)H_{m}}{K_{1}}=\left(2+\frac{K_{2}}{K_{1}}\right)\left(\frac{C_{1m}+C_{2m}}{2}\right).$$

Up to the constant terms, independent on $C_{1}$ and $C_{2}$, Equation (13) is identical to the Helfrich expression for isotropic local bending energy density (Equation (15)) [60,73,90,140,141,142]:

(15) $$w_{b}=\frac{k_{c}}{2}{\left(2H-C_{0}\right)}^{2}+k_{G}K,$$

where $k_{c}$ is the membrane bending constant, $k_{G}$ is the Gaussian bending modulus and $C_{0}$ is the spontaneous curvature of a membrane. In our case it holds: $k_{c}=K_{1}$, $k_{G}=K_{2}$ (see Equations (13) and (15)). The equilibrium local mean curvature for isotropic bending energy (Equation (15)) is determined by requiring $\partial w_{b}/\partial C_{i}=0$, i=1,2, which yields Equation (16) [90]

(16) $$H^{\mathrm{eq}}=\left(C_{1}^{\mathrm{eq}}+C_{2}^{\mathrm{eq}}\right)/2=C_{1}^{\mathrm{eq}}=C_{2}^{\mathrm{eq}}=k_{c}C_{0}/\left(2k_{c}+k_{G}\right)=H_{m}.$$

Microscopic theoretical models for biological membranes [75,90,143] predict that $k_{c}>0$ and $k_{G}<0$, therefore, $K_{1}>0$ and $K_{2}<0$.

In isotropic case membrane contains isotropic (spherical) components: $C_{1m}=C_{2m}=1/R_{m}$, where $R_{m}$ stands for the curvature radius which is favored by the components. Therefore, Equation (14) can be written as (Equation (17)):

(17) $$C_{0}=\left(2+\frac{K_{2}}{K_{1}}\right)\left(\frac{1}{R_{m}}\right).$$

Note that for a special case: $K_{2}=-K_{1}$ (Equation (18)) it holds that (see Equations (14) and (17)):

(18) $$C_{0}=H_{m}=\frac{1}{R_{m}}.$$

This formalism is valid also for the homogeneous isotropic lipid bilayer membrane. Nonzero spontaneous curvature $C_{0}$ in the relaxed state may be expected if the two sides of the lipid bilayer are unequal or facing different aqueous solutions [58]. The shapes of cells/vesicles are therefore likely to be susceptible to chemical agents added to the outer aqueous phase and not instantly permeating to the inner one [58]. For thin and not too strongly curved bilayers, spontaneous curvature could originate from the area difference between the two membrane monolayers [52,56]. If the constituent monolayers are free to slide over each other, $C_{0}$ is expected to be constant over the entire surface area of the cell/vesicle [63].

The total energy of a closed membrane shape (Equation (19)) is equal to the integral of the bending energy density over the whole membrane surface:

(19) $$F_{iso}=\int_{S}w_{b}dS.$$

**Figure 9:**
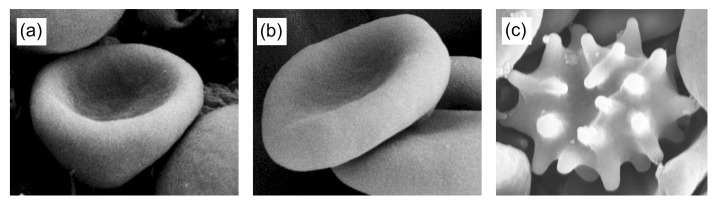
Scanning electron microscope images of red blood cells (RBC) shapes: (**a**) open stomatocyte, (**b**) discocyte, (**c**) echinocyte. Adapted with permission from ref. [137]. 2019 Springer Nature.

By minimizing this energy, it is possible to predict some of the experimentally observed shapes of red blood cells (RBC) like open stomatocytes and discocytes (see Figure 9). To theoretically predict the stable echinocyte shape shown in Figure 9c, one has to take into account also shear deformation of the RBC membrane skeleton, which is described in Section 2.5.1.

### 2.4. Cell Shape Changes Driven by Nematic Orientational Ordering

**Figure 10:**
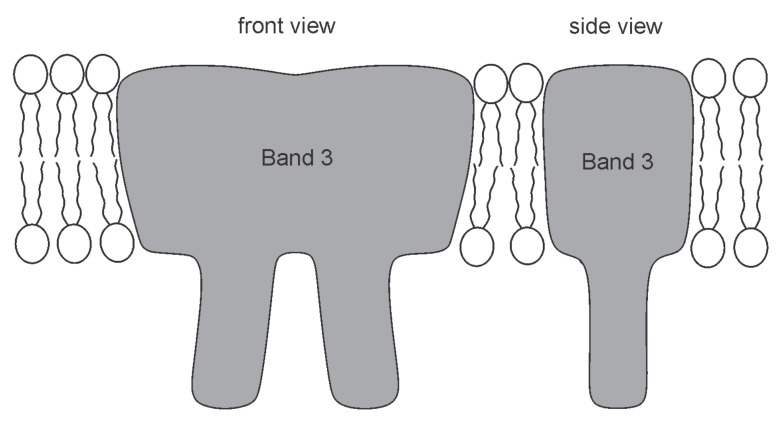
Nematic-type orientational ordering within the membranes of red blood cells or extracellular vesicles could arise due to anisotropic proteins such as Band 3 proteins embedded within membrane. See also Figure 6. Adapted with permission from ref. [137]. 2019 Springer Nature.

Biological membranes very likely possess orientational in-plane ordering, especially in membrane regions that are highly and anisotropically curved [15,79,80]. In-plane ordering could arise for example due to anisotropic Band 3 proteins embedded within membranes (see Figure 10) [79,80,144,145,146]. Taking into account nematic in-plane ordering with direct interactions between anisotropic membrane components could explain the experimentally observed wide stability window of the reduced volume values *v* for stable oblate (discocyte) shapes of red blood cells (RBC). The origin of non-specific attractive nearest-neighbour direct interactions between membrane-embedded proteins, are among others, lipid-mediated depletion- and fluctuation-forces and hydrophobic mismatch between proteins and lipids [147,148,149].

In numerical simulations, the closed membrane surface area *S* and the volume *V* are kept constant. This ensures that an important geometrical parameter, the reduced volume *v*, is also kept constant during numerical minimization. The reduced volume $v=V/V_{0}$ is defined as the ratio of the cell/vesicle volume *V* and the volume of the sphere $V_{0}=4\pi R_{0}^{3}/3$ with the same surface area as the surface of the investigated cell/vesicle, where $R_{0}=\sqrt{S/4\pi }$ is the radius of the sphere and *S* is the surface area of the investigated cell/vesicle shape. All lengths are scaled with respect to $R_{0}$.

The values of *v* in healthy cells (discocyte shape) of different mammals posses a relatively broad range of values [150,151,152,153]. In humans, the experimentally determined reduced volumes of stable discocyte shapes of red blood cells range within the interval 0.58≤v≤0.81 [150], which the existing theoretical approaches are not able to reproduce [46,60,72,73,140,154]. Within the pioneering mesoscopic model introduced by Helfrich [73,140], oblate discocyte shapes are stable only in a relatively narrow window 0.59≤v≤0.65.

**Figure 11:**
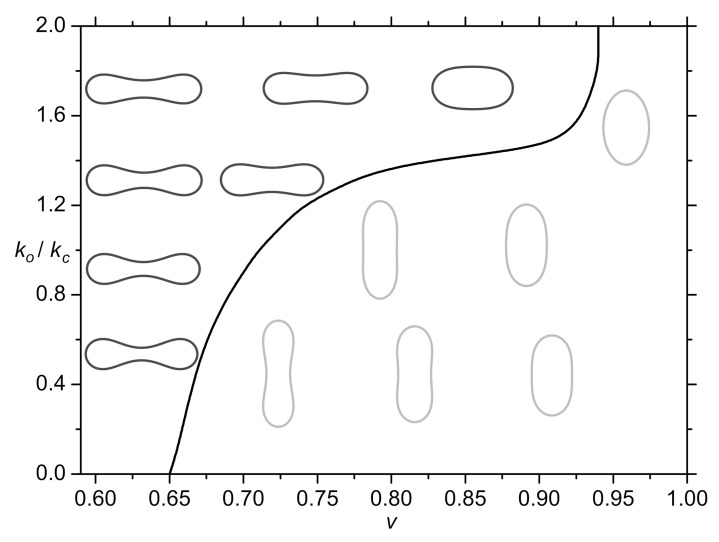
Phase diagram of equilibrium closed membrane shapes with nematic in-plane ordering taken into account. The solid line separates the stability regimes between discocyte (oblate) and prolate shapes. Discocyte shapes are stable on the left side and prolate shapes on the right side of the line. The energy associated with nematic ordering is weighted by the constant $k_{o}$ against the isotropic bending energy weighted by the membrane bending constant $k_{c}$ (Equation (15)). Adapted with permission from ref. [137]. 2019 Springer Nature.

The stability range as a function of the reduced volume *v* is shown in Figure 11 [137]. Equilibrium closed membrane shapes are calculated by minimizing the Helfrich isotropic bending energy (Equation (15)) weighted by the isotropic bending constant $k_{c}$ with the addition of the free energy associated with nematic in-plane ordering (taking into account direct interactions between membrane components). The free energy contributions associated with nematic ordering in the membrane are in this paper described by the constant $k_{o}$. For a detailed description of the nematic ordering energy, see [137] and references therein. If the nematic orientational ordering energy is neglected ($k_{o}<<k_{c}$ in Figure 11), the boundary between oblate (discocyte) and prolate shapes appears at v=0.65. For v>0.65, prolates represent equilibrium stable shapes of RBCs, which is not in agreement with experimental observations where discocyte RBC shapes are found within the interval 0.58≤v≤0.81 [150]. However, when the impact of nematic ordering is increased ($k_{o}\approx k_{c}$), the stability region of stable discocyte shapes widens significantly (Figure 11) [137]. For this phenomenon to occur, one has to take into account the so-called extrinsic (deviatoric) term in orientational ordering energy [137], as described above.

The extrinsic (deviatoric) term tells how the orientational field of molecules is embedded in 3D space [137,155]. The extrinsic-type terms were neglected in many studies so far but in general there is no justification to discard them [156,157]. In studies of biological cells, such and similar terms were considered already in previous studies and referred to as deviatoric terms [15,74,79,80,84,90]. The extrinsic curvature term is effective in points on the membrane surface where the principal curvatures $C_{1}$ and $C_{2}$ differ and its strength increases with the increased absolute value of the curvature deviator $D=(C_{1}-C_{2})/2$ [15,137] (see also Equation (6)). Wide stability range of discocyte shapes in the presence of extrinsic term (Figure 11) is a consequence of their unique shape. In the equatorial region of discocytes, the difference between $C_{1}$ and $C_{2}$ is large so the extrinsic term enforces strong orientational order in that region, which results in the lower total free energy. Consequently, oblate discocyte shapes become more energetically favorable than prolate shapes when the impact of nematic orientational ordering (with the extrinsic term) is taken into account (see Figure 11). Experimentally observed broad stability interval of *v* for discocyte RBC shapes could therefore be explained by considering the orientational in-plane ordering in their membrane [137] (Figure 11).

### 2.5. Influence of Membrane Skeleton

#### 2.5.1. Shear Deformation of Membrane Skeleton and Echinocyte Shape of Red Blood Cells

Red blood cell shape change can be induced by externally added amphiphilic molecules like detergents or peptides that bind into the membrane [53,56,57]. The bilayer couple hypothesis predicts that RBC echinocyte shape transformation is primarily driven by binding of externally added molecules, most probably into the outer membrane layer [45,53,57,74].

Echinocyte shape stability is primarily determined by competition between membrane bending and skeleton shear energy (mentioned already in Equation (15)) [44,45]. The constitutive model for the shear energy of membrane skeleton accounts for its local compressibility [43,158,159,160]. However, an approximate expression for shear energy of the membrane (Equation (20)) is often used due to simplicity [44,161]:

(20) $$F_{shear}=\frac{\mu }{2}\int \left(\lambda_{m}^{2}+\lambda_{m}^{-2}-2\right)dS,$$

where μ is the skeleton area shear modulus of the membrane, $\lambda_{m}$ is the principal extension ratio in the meridional direction and dS is the infinitesimal membrane area element. In this approximation the membrane skeleton is considered laterally incompressible [162].

The total membrane bending energy (Equation (21)) can be written also as the sum of local (Equation (15)) and non-local term [52,56,58,62,161,163]:

(21) $$F_{h}=\frac{k_{c}}{2}\int {\left(2H\right)}^{2}dS+k_{n}S{\left(\langle H\rangle -H_{0}\right)}^{2},$$

where 〈H〉=(1/S)∫HdS is the mean average curvature, $H_{0}$ is the effective mean spontaneous curvature, $k_{c}$ is the membrane isotropic bending constant, $k_{n}$ is the coefficient of non-local bending rigidity [161] and *S* the membrane area.

For thin and only slightly curved bilayers, the mean average curvature 〈H〉 (Equation (22)) is proportional to the difference between the two monolayer areas (ΔS) of the membrane [21,163]:

(22) $$\langle H\rangle =\frac{\Delta S}{2S\delta },$$

where δ is the separation distance between two monolayer neutral surfaces. The normalized average mean curvature $\langle h\rangle =R_{0}\langle H\rangle$ is equal to the normalized area difference $\delta s=\delta S/8\pi \delta R_{0}$, where $R_{0}$ is the radius of the sphere with surface area *S*: $R_{0}=\sqrt{S/4\pi }$.

The normalized effective spontaneous mean curvature $h_{0}=R_{0}H_{0}$ is equal to the normalized optimal area difference $\Delta s_{0}$ (Equation (23)) [26]:

(23) $$h_{0}=\Delta s_{0}=\frac{\Delta S_{0}}{8\pi \delta R_{0}}.$$

The optimal area difference $\Delta S_{0}$ is defined by the difference in the number of constituents (molecules), differences in area of a single molecule and the difference in the intrinsic molecular shapes present in the outer and the inner monolayer (see [26,52,58,62,163] and references therein).

**Figure 12:**
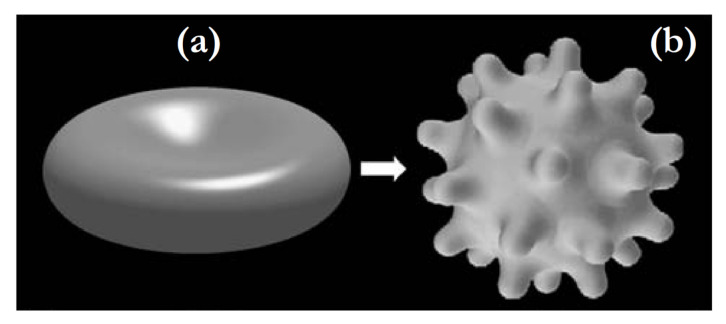
The calculated shapes of erythrocytes determined by minimization of the shear and bending elastic energy (Equations (20) and (21)) for (**a**) $\Delta s_{0}=1.038$ and (**b**) $\Delta s_{0}=6.8$. Furthermore, $k_{n}/k_{c}=8$ [166], $\mu /k_{c}=10^{13}\;m^{-2}$ [161] and the relative cell volume v=0.6. The cell shapes are determined as described in [28] for shape (**a**) and [44,45,164] for shape (**b**). Adapted with permission from ref. [26]. 2007 Elsevier.

Figure 12 shows erythrocyte shapes calculated by minimization of the membrane elastic energy (shear and bending): $F=F_{shear}+F_{h}$ for two different values of optimal area difference $\Delta S_{0}$ [26,44,45,164].

The echinocyte shape can be modulated additionally by stretching the membrane skeleton [43,52] and by a non-homogenous distribution of membrane-embedded proteins [165]. Note that if the membrane skeleton shear elastic energy is neglected, the calculated echinocyte shapes corresponding to a minimum in membrane bending energy (Equation (21)) have only one spiculum [44,45].

#### 2.5.2. Membrane-Myosin Interactions in Red Blood Cells

**Figure 13:**
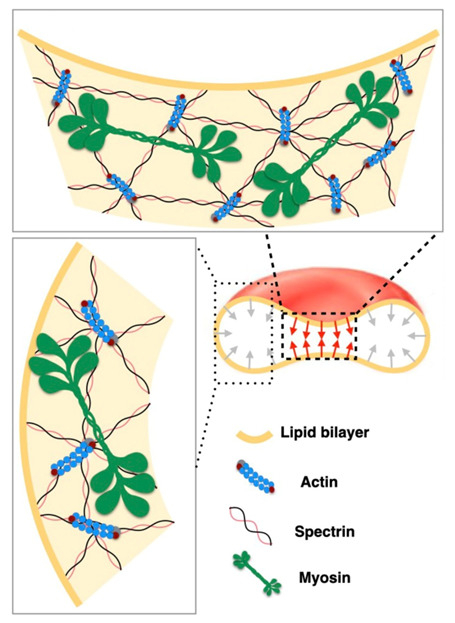
Schematic representation of membrane-myosin interactions in RBC. The shape of the RBC is partially controlled by the interaction of the lipid bilayer and membrane skeleton. Figure shows biconcave discocyte shape of RBC plasma membrane with the membrane skeleton underneath. The myosin (NMIIA) filaments effect (denoted by green colour) was modeled by the local forces applied to the lipid membrane (inward red and gray arrows). Adapted with permission from ref. [49]. 2020 Public Library of Science.

Lacking transcellular cytoskeleton or internal organelles, RBC membrane is supported by the membrane skeleton-a two-dimensional network of short F-actins linked by long, flexible spectrin molecules. These spectrin molecules bind to transmembrane proteins in order to maintain membrane curvature, tension and mechanical properties of the RBC [44,45,46,49]. Furthermore, RBC shape is also influenced by membrane-myosin interactions as schematically shown in Figure 13. The myosin is attached to the spectrin-actin membrane skeleton which may stretch the membrane to additionally stabilize the discocyte RBC shape (Figure 13).

**Figure 14:**
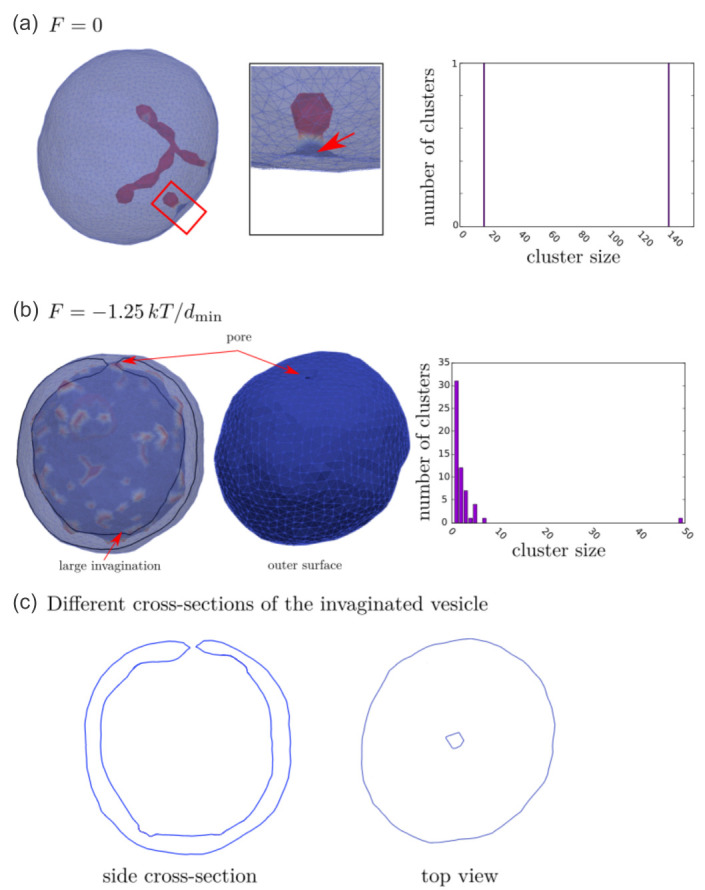
Inset (**a**): The Monte-Carlo simulation of the transformation of the RBC membrane induced by mobile membrane nanodomains with negative local intrinsic curvature $c=-1\;d_{\min}^{-1}$ with the absence of the active force of NMIIA nanodomains. Concentration of membrane nanodomains is p=5%. Semitransparent visualization of the triangulated membrane surface is used in order to uncover its interior shape. Red arrow in the middle enlargement points to the neck area in which there is a lack of nanodomains. In the corresponding cluster-size distributions, the *y*-axis represents the ensemble averaged number of nanodomain clusters of each size, while the *x*-axis is the size of the cluster of inclusions. Other parameters: local bending stiffness of lipid bilayer κ=25kT and direct interaction parameter w=1.25kT. In the inset (**b**), there are results for the same parameters with additional active force of NMIIA nanodomains towards the cell interior. Inset (**c**) shows different views of the vesicle from (**b**) (side cross-section and top view). Adapted with permission from refs. [50,168]. 2020 Slovenian Chemical Society, 2020 Frontiers Media.

In Figure 14, we present MC simulations of a closed membrane shape with isotropic membrane nanodomains of negative intrinsic curvature. The shape of MC predicted closed membrane structures presented in Figure 14 depends on the nanodomain intrinsic curvature, the concentration of nanodomains, the strength of the direct attractive interaction between nanodomains and on the active forces exerted by the nanodomains [50]. Nanodomains with negative intrinsic cvurvature may induce the growth of undulated thin inward membrane protrusions (buds) as demonstrated in Figure 14a. Isotropic membrane nanodomains are accumulated in the region of the protrusions (red colored surface patches in Figure 14a). The theoretically predicted shape in Figure 14a is calculated in the absence of the active force of NMIIA nanodomains and may partially correspond to the situations in RBCs where the protrusion is growing in the region in which the local disruption of the membrane skeleton and the membrane bilayer interactions appears or the skeleton is detached from the protrusion [160,167]. In such cases, the inward membrane protrusion does not contain membrane skeleton [168].

Figure 14a shows also the nanodomains cluster size distributions determined from the averaging over the convergent MC realizations. One observes that the nanodomain cluster size distribution has two distinct peaks corresponding to spheroidal (smaller) and necklace-like (larger) aggregates of nanodomains (Figure 14a). The nanodomains aggregate into curved membrane protrusions or buds as a consequence of non-zero (negative) intrinsic curvature of nanodomains and high enough attractive interaction energy between nanodomains/inclusions [168]. Note that there are no isotropic nanodomains in a highly curved neck region of the invagination (denoted by red arrow in Figure 14a). The membrane curvature in the neck region is anisotropic ($C_{1}\neq C_{2}$) and therefore not favorable for highly curved isotropic ($C_{1}=C_{2}$) nanodomains/inclusions. For long term stabilization of the neck connecting the bud and the parent membrane, one would have to take into account in MC simulations also the effect of anisotropic nanodomains [15,81,89,168,169]. Anisotropic saddle-like nanodomains would in this case assemble in the neck region and stabilize the neck [170].

In simulations presented in Figure 14b, we take into account also the active forces that the membrane NMIIA nanodomains exert in the perpendicular direction to the membrane surface towards the RBC interior. Such active forces in the RBC membrane may be generated by myosin (NMIIA) motor domains bound to F-actin of the RBC membrane skeleton [48] (see also Figure 13). We observe in Figure 14b that in the case where membrane nanodomains are exerting force on the membrane, the MC predicted RBC shape has one large invagination [50], which is in agreement with some experiments [54,55]. Cluster size distribution in Figure 14b shows that the membrane contains many smaller aggregates of nanodomains. For visualization purposes, different views of the invaginated vesicle from Figure 14b are presented in Figure 14c.

Note that large invaginations can be separated from the parent cell due to local frustrations in orientational in-plane ordering of membrane constituents in highly curved membrane parts such as membrane necks connecting the invagination and the parent cell [168,171]. Highly curved membrane regions such as membrane necks are likely to host topological defects, which are a source of large elastic penalties [168,171]. Invaginated stomatocyte neck region is a relatively small surface, which can host up to four topological defects. Consequently, within the neck region, local interactions between neighboring molecules are weakened, which might lead to the neck rupture [168,171].

Figure 14 demonstrates that invaginated stomatocytic RBC shapes can have spherical or undulated necklace-like invaginations. Furthermore, MC simulations in [50] reveal that a larger concentration of NMIIA nanodomains exerting force on the membrane can induce also pancake-like torocyte membrane invaginations, which were observed in some experiments [172]. These results represent an extension of the previous theoretically predicted invaginated stomatocyte shape classes, which were limited mostly to the simple axisymmetric stomatocyte with only one large invagination [50].

Note that in MC simulations [24,50,168] presented in Figure 14, the bilayer structure of the membrane and the skeleton elasticity are not explicitly considered. Consequently, the value of the bending modulus is set to be compatible with the membrane of giant lipid vesicles [173,174] and not with the RBC membrane [47,175,176,177,178]. Furthermore, for simplicity, only one type of nanodomains/inclusions that can induce local bending of the membrane by cause of their negative intrinsic curvature is considered in MC simulations [24,50,168].

### 2.6. Theory of Self-Assembly of Isotropic Curved Membrane Components into Larger Domains

**Figure 15:**
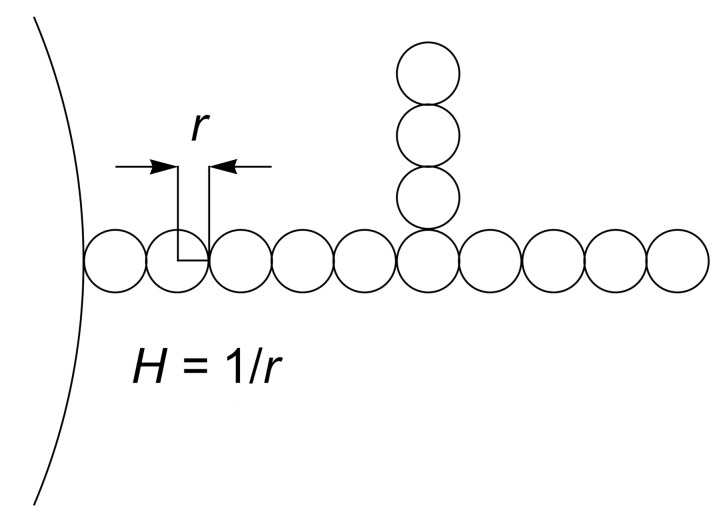
Growth of necklace-like protrusions as aggregates of isotropic curved membrane nanodomains is energetically favorable when critical concentration ${\tilde{x}}_{c}$ of nanodomains is surpassed.

Theory of self-assembly may be used to describe the accumulation of isotropic curved membrane nanodomains (Figure 6) composed of lipids and proteins [12,22,26] into spherical or necklace membrane protrusions. In the beginning, curved nanodomains (of total number *N*) are distributed in the weakly curved spherical membrane surface of constant mean curvature $H=1/R_{0}$. We assume that the curved nanodomains are laterally mobile across the whole membrane surface and may at certain conditions form highly curved aggregates in the form of curved membrane protrusions of constant high mean curvature H=1/r. The membrane protrusion with constant H=1/r which may be a single sphere or necklace formation (see Figure 15). We assume $r\ll R_{0}$.

In the following we determine the critical concentration of isotropic curved nanodomains necessary for formation of neklace-like protrusions of the membrane in the self-assembly process. For the sake of simplicity we presume that the free energy of a single isotropic flexible membrane nanodomain (Equation (24)) (Figure 6) is written in the form [12,22,26,84]:

(24) $$f=\frac{\xi }{2}{\left(H-H_{m}\right)}^{2}a_{0}\;.$$

where $H_{m}$ is the intrinsic mean curvature of an isotropic membrane nanodomain, $\xi =4K_{1}+2K_{2}$ is the elastic constant (see Equation (13)) and $a_{0}$ is the area per single nanodomain. In large aggregates of curved flexible membrane nanodomains the local membrane bending constant is $k_{c}=K_{1}$ (Equation (13)) and the membrane spontaneous curvature $C_{0}=\left(2K_{1}+K_{2}\right)H_{m}/K_{1}$ (Equation (14)).

Curved flexible membrane nanodomains in aggregates interact with neighboring membrane nanodomains. We denote the interaction energy per curved flexible membrane nanodomain (monomer) in an aggregate composed of *i* nanodomains as w(i) where we presume that the energy w(i) depends on aggregate size consisting of *i* nanodomains. The mean free energy per nanodomain in a curved aggregate (where H=D=1/r) composed of *i* nanodomains is written as (Equation (25)):

(25) $$\mu_{i}=f_{c}-w\left(i\right)\;,$$

where $f_{c}=f(H=1/r)$ and w(i)>0. We assume that in the weakly curved spherical regions of the membrane (having $H=1/R_{0}$) nanodomain concentration is always below the critical aggregation concentration and nanodomains cannot form 2D flat aggregates. The mean energy of a nanodomain in the weakly curved membrane regions is ${\tilde{\mu }}_{1}=f_{sp}\;$, where $f_{sp}=f\left(H=1/R_{0}\right)$. The number density of curved nanodomains in the weakly curved membrane regions is (Equation (26))

(26) $${\tilde{x}}_{1}=\frac{{\tilde{N}}_{1}}{M}\;,$$

where ${\tilde{N}}_{1}$ is the number of monomeric curved nanodomains in the weakly curved membrane regions and *M* is the lattice sites number in the whole system. The distribution of highly curved aggregates of nanodomains in the membrane protrusions on the scale of number density (Equation (27)) is expressed as

(27) $$x_{i}=\frac{iN_{i}}{M}\;,$$

where $N_{i}$ represents the number of aggregates with *i* constituents. The number densities ${\tilde{x}}_{1}$ and $x_{i}$ must fulfil the conservation condition for the total number of curved flexible nanodomains (Figure 6) in or on the membrane (Equation (28)):

(28) $${\tilde{x}}_{1}\;+\sum_{i=1}^{\infty }x_{i}=N/M\;.$$

The free energy *F* of all curved nanodomains in or on the membrane is (Equation (29)):

(29) $$\begin{array}{c} F=M\left[{\tilde{x}}_{1}\;{\tilde{\mu }}_{1}+kT{\tilde{x}}_{1}\left(\ln{\tilde{x}}_{1}-1\right)\right]+M\sum_{i=1}^{\infty }\left[x_{i}\;\mu_{i}+kT\frac{x_{i}}{i}\left(\ln\frac{x_{i}}{i}-1\right)\right]-\\ -\mu M\;({\tilde{x}}_{1}+\sum_{i=1}^{\infty }x_{i})\;\;, \end{array}$$

where μ is the Lagrange parameter ensuring a constant number of curved nanodomains in the system. Contributions of configurational entropy are also taken into account in the free energy. We minimize *F* with respect to ${\tilde{x}}_{1}$ and $x_{i}$ (Equation (30)):

(30) $$\begin{array}{c} \frac{\partial F}{\partial {\tilde{x}}_{i}}=0\;,\frac{\partial F}{\partial x_{i}}=0,\;i=1,\;2,\;3,\;\ldots \;, \end{array}$$

which leads to equilibrium distributions (Equations (31) and (32)):

(31) $${\tilde{x}}_{1}=\exp\left(-\;\frac{f_{sp}-\mu }{kT}\right)\;,$$

(32) $$x_{i}=i\;\exp\left(-\frac{i}{k\;T}\;\left[f_{c}-w-\mu \right]\right)\;.$$

**Figure 16:**
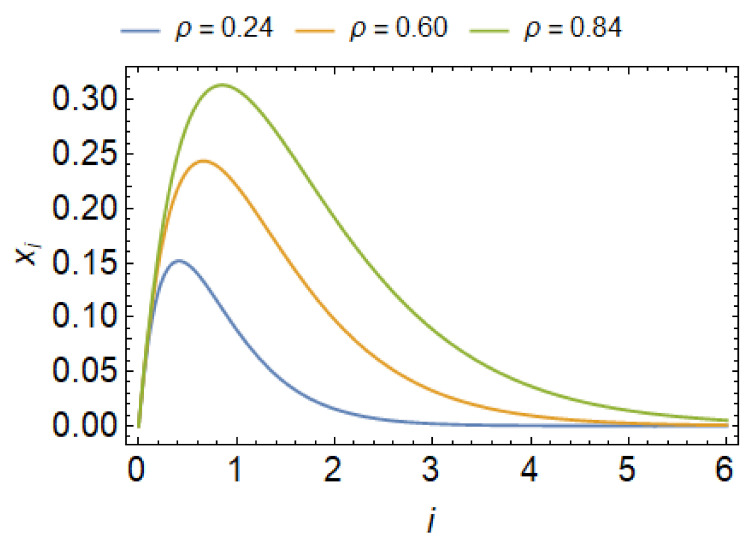
An example of concentrations of aggregates of nanodomains in dependence on number of nanodomains in the aggregate for different number of flexible nanodomains on the membrane.

This dependence $x_{i}\left(i\right)$ is shown in Figure 16 for different values of total number of flexible nanodomains on the membrane. For simplicity we assumed that w(i)=w is independent of aggregate size in Equation (32). The quantity μ can be expressed from Equation (31) and substituted in Equation (32) to get (Equation (33)):

(33) $$x_{i}=i\;{\left[{\tilde{x}}_{1}\cdot \exp\left(\frac{f_{sp}+w-f_{c}}{kT}\right)\right]}^{i}\;.$$

We see that if the concentration ${\tilde{x}}_{1}$ is small, aggregate growth will not be favorable, since $x_{1}>x_{2}>x_{3}\;\ldots$. Furthermore, $x_{i}$ can never exceed unity, leading to the largest possible value of monomeric curved flexible nanodomains number density in the weakly curved parts of the membrane when ${\tilde{x}}_{1}$ approaches $\exp\left[(f_{c}-f_{sp}-w)/k\;T\right]$. The critical concentration is therefore (Equation (34))

(34) $${\tilde{x}}_{c}\approx \exp\left(\;\frac{\Delta f-w}{kT}\right)\;,$$

where $\Delta f=f_{c}-f_{sp}$ is the energy difference between a single nanodomain on the highly curved membrane protrusion and the energy of the single nanodomain in the weakly curved membrane region with (Equation (35)):

(35) $$\Delta f=\frac{\xi a_{0}}{2r}\left(\frac{1}{r}-2H_{0}\right)-\frac{\xi a_{0}}{2R_{0}}\left(\frac{1}{R_{0}}-2H_{0}\right)\;.$$

**Figure 17:**
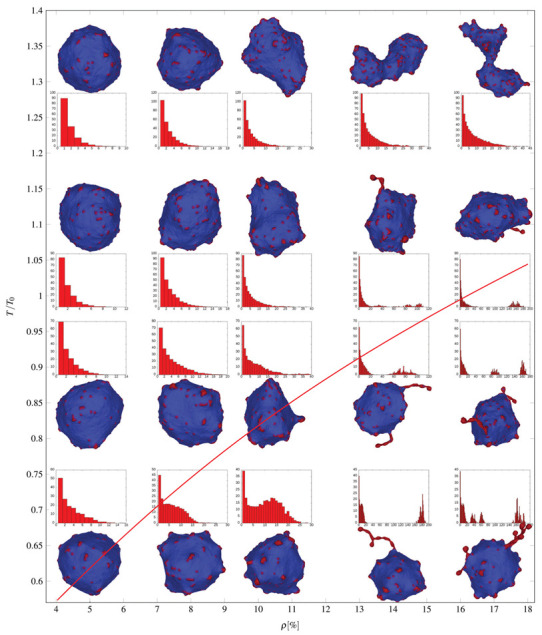
Monte-Carlo (MC) predicted vesicle shapes in thermal equilibrium with curved flexible nanodomains. The diagram provides information for differing average densities of curved nanodomains ρ and relative temperatures $T/T_{0}$. Blue surfaces represents the nanodomain-free lipid bilayer and has zero intrinsic (spontaneous) curvature; red surface denotes the curved nanodomain clusters with spontaneous curvature $c_{0}$. In the corresponding cluster-size distributions below each snapshot, the *y*-axis is the ensemble averaged number of nanodomain clusters and the *x*-axis is the nanodomain cluster size. The red curve is a boundary between shapes without and with protrusions predicted by the theory of self-assembly. Below it, growth of membrane protrusions is favorable (see Equation (32)). Adapted with permission from ref. [24]. 2019 Royal Society of Chemistry.

Critical aggregation number density is given by ${\tilde{x}}_{c}$. If ${\tilde{x}}_{1}$ is larger than ${\tilde{x}}_{c}\;$, the formation of a very long necklace membrane protrusions composed of curved membrane nanodomains is energetically favorable. This is also observed in our MC simulations (Figure 17). It can be seen from Equation (34) that growth of necklace membrane protrusions is dependent on the energy difference Δf (Equation (35)) and the strength of the direct nearest-neighbor interaction between nanodomains *w*. The critical concentration ${\tilde{x}}_{c}$ strongly depends on $H_{0}$.

In the approximation limit $R_{0}\gg r$ we can rewrite Equation (35) as (Equation (36)):

(36) $$\Delta f≃\frac{\xi a_{0}}{2r}\left(\frac{1}{r}-2H_{0}\right)=\frac{2k_{c}a_{0}}{r}\left(\frac{1}{r}-c_{0}\right)\;,$$

where $k_{c}$ and $c_{0}$ are the local bending constant and spontaneous curvature of aggregates of nanodomains, respectively. We may rewrite Equation (34) as (Equation (37)):

(37) $${\tilde{x}}_{c}\approx \exp\left(\;2\;\frac{k_{c}}{kT}\;\frac{a_{0}}{r^{2}}\left(1-c_{0}r\right)-\frac{w}{kT}\right)\;.$$

For $1<c_{0}r$ the value of Δf is always negative.

In the case of anisotropic curved nanodomains, Equation (34) still holds, while in the case of cylindrical anisotropic aggregates, where H=D=1/2r, Δf (Equation (38)) is (see [179])

(38) $$\Delta f=a_{0}\xi H\left(H-H_{m}\right)-kT\ln\left[I_{0}\left(\frac{a_{0}\xi D_{m}D}{kT}\right)\right].$$

Here, $H=(C_{1}+C_{2})/2$ is the mean curvature of the membrane, the intrinsic mean curvature of an isotropic membrane nanodomain is $H_{m}=\left(C_{1m}+C_{2m}\right)/2$, $D=(C_{1}-C_{2})/2$ is the curvature deviator of the membrane, and $D_{m}=\left(C_{1m}-C_{2m}\right)/2$ is the intrinsic curvature deviator of the membrane element and $I_{0}$ represents the modified Bessel function [15,21,74,77,78,79,80,83,90,179].

The theory of self-assembly can be used to predict phase space separations in diagrams of MC simulated equilibrium vesicle shapes with curved nanodomains. Figure 17 shows a plot of typical microstates of vesicles with curved nanodomains in the absence of active protrusive forces. The vesicles are in thermal equilibrium at different temperatures and nanodomain densities (area coverage fraction, $\rho =N_{c}/N$) of curved nanodomains. The cluster size distributions (shown below the microstate snapshots in Figure 17) are given from averaging over convergent MC shapes.

At low average nanodomain area densities the equilibrium vesicle morphologies remain quasi-spherical, with clusters that increase in size at lower temperatures (in the far left column of Figure 17, the largest clusters consist of 5 nanodomains at $T/T_{0}$ = 1.33 and of 8 nanodomains at $T/T_{0}$ = 0.63). At higher average nanodomain densities, clusters increase in size and curved nanodomain buds appear on the membrane.

At even larger average nanodomain densities, the vesicle shapes drastically deviate from quasi-spherical morphologies, often forming large necklace-like nanodomain clusters. The size of these clusters of necklace-like nanodomains and the number of ’beads’ they consist of increases with decreasing temperature. These necklace-like structures form because isotropic curved nanodomains cannot form flat aggregates, due to their high intrinsic curvature. The theory of self-assembly of curved nanodomains (Figure 6) can approximately explain the basic principles of formation of observed necklace-like structures.

Since the density of curved nanodomains (Figure 6) in or on the membrane is defined with the conservation condition (Equation (28)), this also gives us the relation between normalized temperature $T/T_{0}$ and total curved nanodomains concentrations ρ=N/M. Using the parameters from the MC simulations, we may graph dependencies $x_{i}\left(i\right)$, as seen in Figure 17. For sufficiently low concentrations ${\tilde{x}}_{1}$, such that ${\tilde{x}}_{1}\cdot \exp\left[\left(f_{sp}+w-f_{c}\right)/kT\right]$ is much less than unity, we have $x_{1}>x_{2}>x_{3},\ldots$, which means that most of the nanodomains will be found in the weakly curved membrane region. Above small concentrations and especially above ${\tilde{x}}_{c}$, aggregates start to form, where the peaks of the distributions are strongly dependent on the total nanodomain concentration in the lattice. We see that the critical line beyond which aggregate growth is favorable agrees well with the results of MC simulations (see red line in Figure 17).

**Figure 18:**
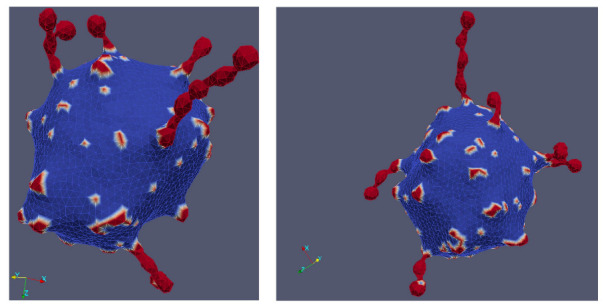
A typical vesicle shape calculated by MC simulations for two-component membrane composed of highly curved isotropic flexible nanodomains (red color) and the nanodomains with zero intrinsic curvature (blue color). Membrane nanodomains with high intrinsic curvature (red color) are accumulated in undulated membrane protrusions. Adapted with permission from ref. [23]. 2017 Springer Nature.

The distribution of cluster sizes features a second peak below the transition line, corresponding to the formation of large aggregates of curved nanodomains (Figure 17). For low temperatures and large nanodomain densities, the distributions of necklace-like aggregates is predicted to be of an exponential nature (Equation (34)). However, due to small vesicle size and the simulations getting ‘‘stuck’’ in particular aggregate configurations, this feature is not observed in distributions. A close-up snapshot of a MC predicted vesicle shape with a few formed beaded protrusions consisting of highly curved flexible isotropic nanodomains can be seen in Figure 18.

### 2.7. Free Energy of Two-Component Anisotropic Membrane: An Approximative Approach

For the simplest case, where we assume that the principal systems of the actual local membrane curvature tensor $\underline{C}$ and the intrinsic membrane curvature tensor ${\underline{C}}_{m}$ coincide everywhere on the surface (ω=0 in Equation (6)), the elastic energy density transforms into (Equation (39)) [77,90]:

(39) $$w=\left(2K_{1}+K_{2}\right){\left(H-H_{m}\right)}^{2}-K_{2}{\left(D-D_{m}\right)}^{2}.$$

The equilibrium values of *H* and *D* (and consequently $C_{1}$ and $C_{2}$) corresponding to the extreme of the function *w* (determined from $\partial w/\partial C_{i}=0$, i=1,2) are (Equation (40)) [90]

(40) $$H^{\mathrm{eq}}=H_{m},D^{\mathrm{eq}}=D_{m}.$$

If the above condition is met, membrane component perfectly fits into the membrane surface. Equilibrium values of *H* and *D* (Equation (40)) correspond to the local minimum of *w* if the local stability condition (Equation (41)) for a completely free and small patch of the membrane is true [90]:

(41) $$\left[\left(\frac{\partial^{2}w}{\partial {C_{1}}^{2}}\right)\left(\frac{\partial^{2}w}{\partial {C_{2}}^{2}}\right)-{\left(\frac{\partial^{2}w}{\partial C_{1}\partial C_{2}}\right)}^{2}\right]>0,$$

which yields (Equation (42)):

(42) $$-K_{2}\left(2K_{1}+K_{2}\right)>0.$$

Considering the isotropic limit, it was shown in Section 2.3 that $k_{c}=K_{1}$ and $k_{G}=K_{2}$. The results of microscopic theoretical biological membrane models [75,90,143] predict that the Helfrich’s constants $k_{c}$ and $k_{G}$ are of the same order of magnitude and that $k_{c}>0$ and $k_{G}<0$. We can therefore assume that $K_{1}>0$ and $K_{2}<0$, which is in agreement with the above stability condition (see Equation (42)): $(2K_{1}+K_{2})>0$, i.e., $K_{1}>-K_{2}/2$. Based on these results, we assume in the following that $K_{2}\approx -K_{1}$ [90], which simplifies Equation (39) to the form (Equation (43)):

(43) $$w=K_{1}{\left(H-H_{m}\right)}^{2}+K_{1}{\left(D-D_{m}\right)}^{2}.$$

In the following, we consider a membrane that is composed of two different nanodomains/components *A* and *B*, characterized by the intrinsic principal curvatures $C_{1m}^{i}$ and $C_{2m}^{i}$, where i=A,B. Note that curved flexible nanodomains *A* and *B* can be isotropic or anisotropic (see Figure 6 and Figure 7). The bending energy of the whole two-component membrane (Equation (44)) is calculated by integration of *w* (Equation (43)) over the whole membrane area *S* [23,89,90,180]:

(44) $$F_{b}=\int_{S}k_{c}\left(\phi \right)\left[{\left(H-H_{m}\left(\phi \right)\right)}^{2}+{\left(D-D_{m}\left(\phi \right)\right)}^{2}\right]dS,$$

where dS is an infinitesimal element of the membrane surface area *S* and ϕ is the local relative area density of the component *A*. The local relative area density of the component *B* is therefore (1−ϕ). Constant $K_{1}$ from Equation (43) was replaced with the bending rigidity constant $k_{c}\left(\phi \right)$, which depends on ϕ because in general, different membrane nanodomains (in our case components *A* and *B*) can have different bending rigidities. It can be assumed that the bending rigidity $k_{c}\left(\phi \right)$ (Equation (45)), the intrinsic mean curvature $H_{m}\left(\phi \right)$ (Equation (46)) and the curvature deviator $D_{m}\left(\phi \right)$ (Equation (47)) depend linearly on the local relative area density of nanodomains *A* (ϕ):

(45) $$k_{c}\left(\phi \right)=\left(\kappa^{A}-\kappa^{B}\right)\phi +\kappa^{B},$$

(46) $$H_{m}\left(\phi \right)=\left(H_{m}^{A}-H_{m}^{B}\right)\phi +H_{m}^{B},$$

(47) $$D_{m}\left(\phi \right)=\left(D_{m}^{A}-D_{m}^{B}\right)\phi +D_{m}^{B},$$

where $\kappa^{i}$ is the bending rigidity of the component *i*, $H_{m}^{i}=\left(C_{1m}^{i}+C_{2m}^{i}\right)/2$ is the intrinsic mean curvature of the component *i* and $D_{m}^{i}=\left(C_{1m}^{i}-C_{2m}^{i}\right)/2$ is the intrinsic curvature deviator of nanodomains *i*, where i=A,B. Membrane nanodomains *i* are considered isotropic when their intrinsic deviatoric curvature $D_{m}^{i}=0$ ($C_{1m}^{i}=C_{2m}^{i}$).

For two-component membrane we should take into account also the entropy of mixing of these two components (Equation (48)). The second part of the membrane free energy is therefore associated with the entropy of mixing [25,89,180]:

(48) $$F_{mix}=\frac{k_{B}T}{a_{0}}\int_{S}\left[\phi \ln\phi +(1-\phi )\ln\left(1-\phi \right)\right]dS,$$

where $k_{B}$ is the Boltzmann constant, *T* is the absolute temperature and $a_{0}$ is the area of a single nanodomain of type *A* or *B*. The free energy functional of such two-component membrane (Equation (49)) is the sum of the energy contributions defined by Equations (44) and (48):

(49) $$F=F_{b}+F_{mix}.$$

**Figure 19:**
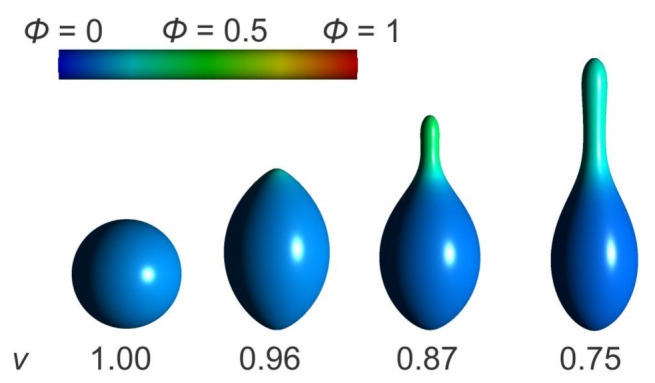
Equilibrium closed membrane shapes calculated for different values of the reduced volume *v*. The entropy of mixing was taken into account in minimization of the membrane free energy. Membrane contains two types of nanodomains denoted by *A* (anisotropic) and *B* (isotropic). Fixed value of average relative area density is $\phi_{ave}=0.15$ for the nanodomains *A* and $\left(1-\phi_{ave}\right)$ for the nanodomains *B*. The red color (ϕ=1) represents the highest possible local relative area density (concentration) of the nanodomains *A*, while the local concentration of the nanodomains *B* is given by (1−ϕ). Shapes were calculated by minimizing the energy functional given by Equation (49) for the following values of model parameters: $H_{m}^{B}=1$, $D_{m}^{B}=0$, $H_{m}^{A}=8$, $D_{m}^{A}=8$, $\kappa^{A}=8\;\kappa^{B}$, $\kappa^{B}=30\;k_{B}T$, $R_{0}=250\;\mathrm{nm}$ and $a_{0}=100\;{\mathrm{nm}}^{2}$. Here, $R_{0}$ is the radius of the sphere with the same surface area as the surface of the investigated cell/vesicle. Adapted with permission from ref. [23]. 2017 Springer Nature.

Figure 19 shows axisymmetric vesicle shapes calculated for different values of the reduced volume *v* by minimizing the energy functional given by Equation (49). The average relative area density of anisotropic nanodomains *A* is $\phi_{ave}=0.15$, while the remaining surface area of the closed membrane shape is covered by isotropic nanodomains *B*. According to the definition of the reduced volume, for v=1, the vesicle can only be in a spherical shape. In this case, both types of nanodomains are homogeneously mixed throughout the surface (Figure 19). As the value of the reduced volume gets lower, different membrane nanodomains start to accumulate into separate regions (Figure 19). The entropy contribution to the free energy functional $F_{mix}$ (Equation (48)) enforces different kinds of membrane nanodomains to intermix, for which reason the lateral segregation of the isotropic and anisotropic nanodomains is relatively weak. Nevertheless, anisotropic nanodomains enforce the formation of a tubular protrusion for v=0.87 and v=0.75 in Figure 19 [23].

**Figure 20:**
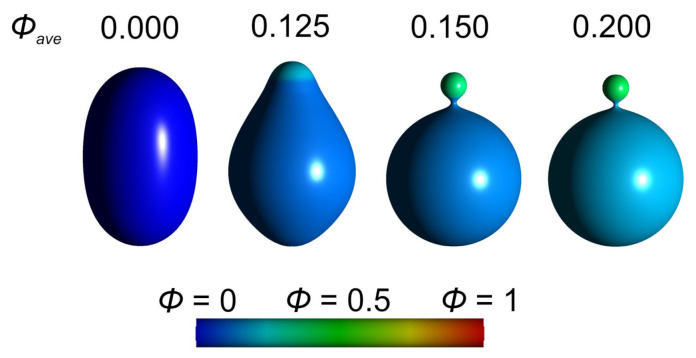
Equilibrium closed membrane shapes calculated for different values of the average relative area density $\phi_{ave}$ of highly curved isotropic nanodomains *A*. Membrane contains two types of flexible isotropic nanodomains denoted by *A* (highly curved) and *B* (slightly curved). The average relative area density of nanodomains *B* is $\left(1-\phi_{ave}\right)$. The red color (ϕ=1) represents the highest possible local relative area density (concentration) of the nanodomains *A*, while the local concentration of the nanodomains *B* is given by (1−ϕ). The entropy of mixing was taken into account in minimization of the membrane free energy. Shapes were calculated by minimizing the energy functional given by Equation (49) for the following values of model parameters: v=0.95$H_{m}^{B}=1$, $D_{m}^{B}=0$, $H_{m}^{A}=16$, $D_{m}^{A}=0$, $\kappa^{A}=8\;\kappa^{B}$, $\kappa^{B}=30\;k_{B}T$, $R_{0}=250\;\mathrm{nm}$ and $a_{0}=100\;{\mathrm{nm}}^{2}$. Here, $R_{0}$ is the radius of the sphere with the same surface area as the surface of the investigated cell/vesicle. Adapted with permission from ref. [170]. 2020 Public Library of Science.

In Figure 20 we demonstrate the formation of an isotropic bud as a consequence of highly curved isotropic nanodomains. As the average relative area density $\phi_{ave}$ of highly curved isotropic flexible nanodomains *A* is increased, a small spherical bud is formed. We observe some degree of lateral segregation of the nanodomains *A* and *B*, i.e., the local relative area density ϕ (concentration) of highly curved nanodomains *A* is increased in the small spherical bud and decreased in the remaining part of the vesicle (Figure 20).

**Figure 21:**
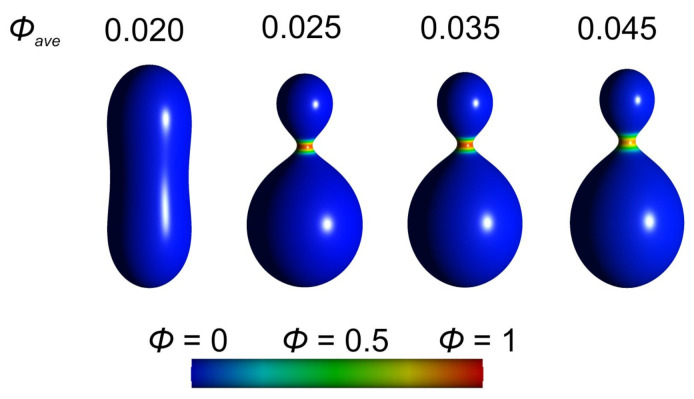
Equilibrium closed membrane shapes calculated for different values of the average relative area density $\phi_{ave}$ of anisotropic saddle-like nanodomains *A*. Membrane contains two types of nanodomains denoted by *A* (saddle-like anisotropic) and *B* (isotropic). The average relative area density of nanodomains *B* is $\left(1-\phi_{ave}\right)$. The red color (ϕ=1) represents the highest possible local relative area density (concentration) of the nanodomains *A*, while the local concentration of the nanodomains *B* is given by (1−ϕ). The entropy of mixing was taken into account in minimization of the membrane free energy. Shapes were calculated by minimizing the energy functional given by Equation (49) for the following values of model parameters: v=0.8$H_{m}^{B}=2$, $D_{m}^{B}=0$, $H_{m}^{A}=0$, $D_{m}^{A}=8$, $\kappa^{A}=8\;\kappa^{B}$, $\kappa^{B}=30\;k_{B}T$, $R_{0}=250\;\mathrm{nm}$ and $a_{0}=100\;{\mathrm{nm}}^{2}$. Here, $R_{0}$ is the radius of the sphere with the same surface area as the surface of the investigated cell/vesicle. Adapted with permission from ref. [170]. 2020 Public Library of Science.

The effect of anisotropic saddle-like nanodomains is shown in Figure 21. As the average relative area density $\phi_{ave}$ of anisotropic saddle-like nanodomains *A* is increased, an anisotropic neck connecting two parts of the vesicle is formed. We observe a high degree of lateral segregation of nanodomains *A* and *B*. Almost all saddle-like anisotropic nanodomains *A* are accumulated within the neck region, while the remaining surface area of the cell/vesicle is covered with slightly curved isotropic components *B* (Figure 21).

## 3. Cytoskeleton and Cell Shape

### 3.1. Interplay between Cytoskeleton Force and Distribution of Curved Membrane Nanodomains in Membrane Protrusive Growth

**Figure 22:**
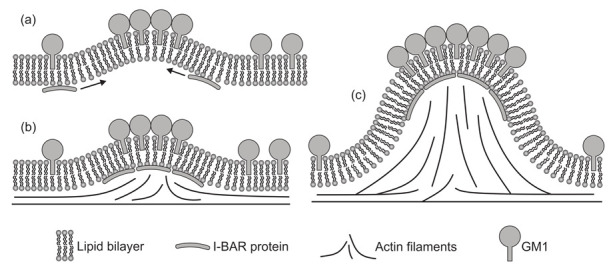
Schematic diagram of the possible mechanism of the membrane protrusion growth. (**a**) GM1 aggregates/nanodomains with positive intrinsic curvature and I-BAR protein domains generate the initial plasma membrane outward deformation. (**b**) More I-BAR domains are attracted to the negative curvature region at the inner leaflet of the membrane. They accumulate and partially support the initial protrusion. Actin filaments start to fill the space inside the initial protrusion through stochastic polymerization. (**c**) I-BAR domain proteins bend the membrane further, while actin filaments are filling the space inside the protrusion. The nucleation of actin filaments drives the membrane protrusive growth, elongating or stabilizing the membrane protrusion. Adapted with permission from ref. [71]. 2011 Dove Press.

We discuss the effect of actin cytoskeleton mechanical force on closed membrane shapes such as cells and vesicles. Our main goal is to analyze the membrane tubular structures generated by such force. A possible mechanism of membrane protrusion growth is demonstrated in Figure 22. The initial membrane deformation may be generated by GM1 aggregates and I-BAR protein domains. GM1s themselves have a curvature-generating mechanism when they are close to each other (Figure 22) [71]. Furthermore, they may also have an indirect impact through the recruitment of I-BAR protein domains, which favor negative curvature [18], initially generated by GM1s. It has been shown that I-BARs, bound to the inner membrane leaflet, can generate a negative membrane curvature (which in turn recruits more I-BAR domains [121]) and mediate the actin nucleation machinery [71,122,181,182]. The attached I-BAR domains may induce actin self-assembly inside the protrusion and in this way promote the elongation or stabilization of the membrane nanotubes [18,71,94]. The space inside the membrane protrusion is filled by actin filaments in the process of stochastic polymerization [93]. It is not fully clear if the membrane protrusions are pushed and deformed by the polymerizing actin filaments, or they are only stabilized by actin filaments [18,93]. In any case, actin cytoskeleton is required for long term stabilization of the membrane protrusions [93].

**Figure 23:**
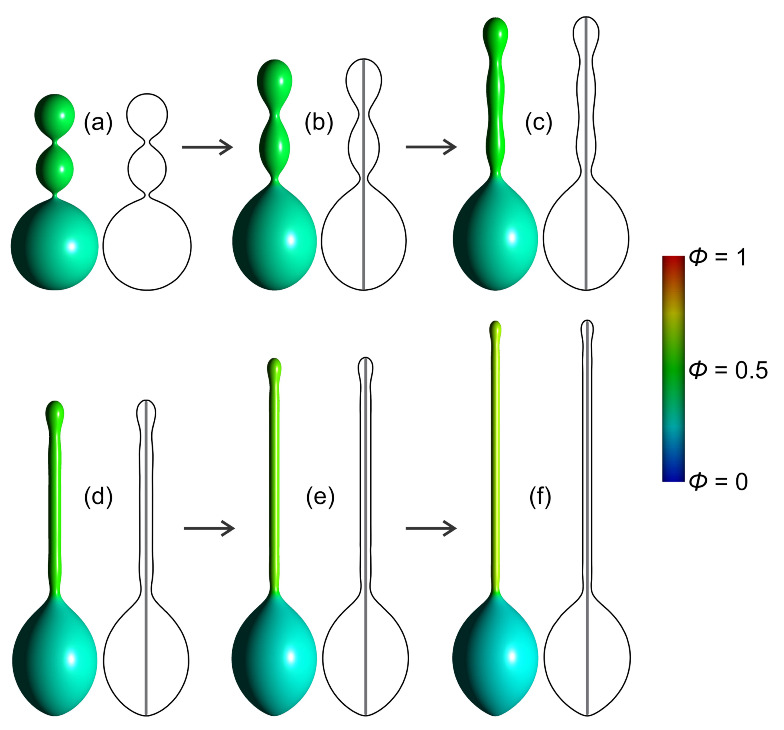
Equilibrium closed membrane shapes calculated for different lengths of the cytoskeleton rod-like structure inside the membrane (schematically shown as a grey rod inside the profile curve for each shape). Membrane contains two types of nanodomains denoted by *A* and *B*. Fixed value of average relative area density is $\phi_{ave}=0.35$ for the nanodomains *A* and $\left(1-\phi_{ave}\right)$ for the nanodomains *B*. The red color (ϕ=1) represents the highest possible local relative area density (concentration) of curved nanodomains *A*, while the local concentration of flat nanodomains *B* is given by (1−ϕ). For simplicity reasons, the entropy of mixing of different membrane nanodomains was not taken into account. Shapes were calculated within the model introduced in Section 2.7 for the following values of model parameters: v=0.70, $H_{m}^{B}=0$, $D_{m}^{B}=0$, $H_{m}^{A}=12$, $D_{m}^{A}=0$, $\kappa^{A}=\kappa^{B}$.

**Figure 24:**
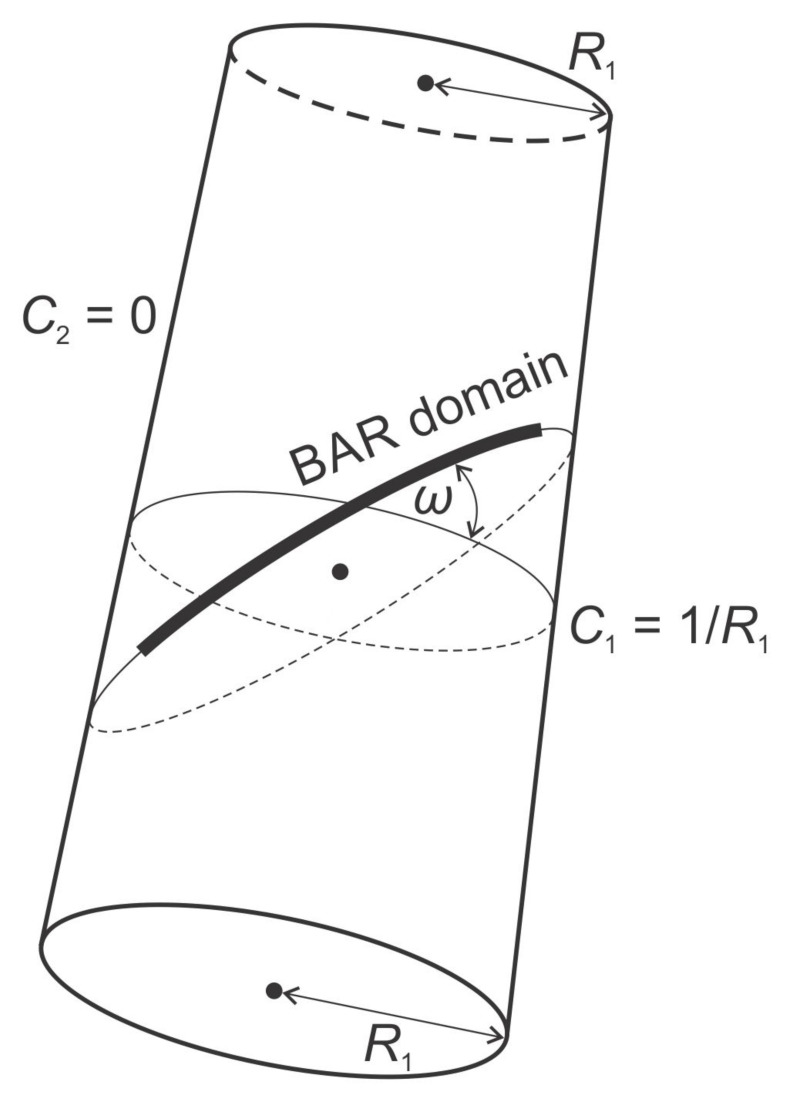
Schematic presentation of the single rod-like BAR domain attached to the cylindrical surface, where $R_{1}$ is the radius of the cylinder. Angle ω is the angle of the normal plane in which the BAR domain is lying relative to the normal plane of the first principal curvature $C_{1}$. $C_{2}$ is the second principal curvature of the cylinder. Adapted with permission from ref. [18]. 2016 Elsevier.

**Figure 25:**
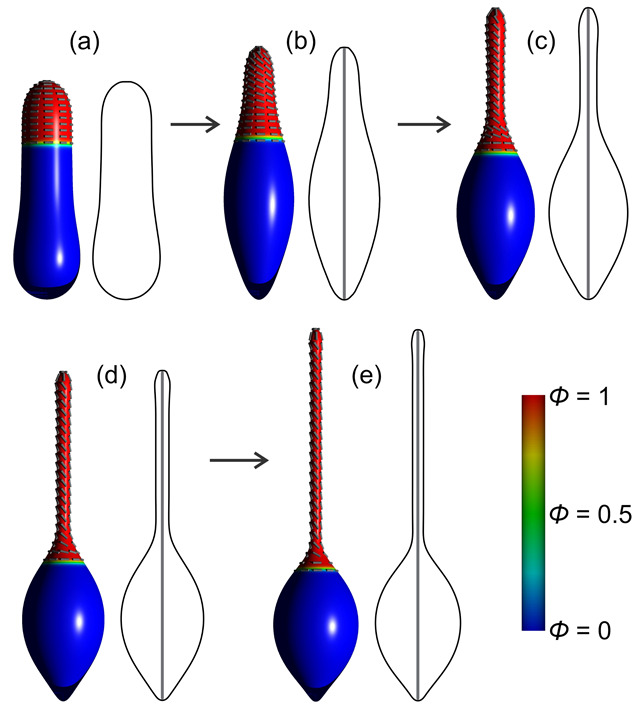
Equilibrium closed membrane shapes calculated for different lengths of the cytoskeleton rod-like structure inside the membrane (schematically shown as a grey rod inside the profile curve for each shape). Membrane contains a fixed value of average relative area density (concentration) of anisotropic rod-like BAR domains $\phi_{ave}=0.25$. The red color (ϕ=1) represents the highest possible local relative area density (concentration) of BAR domains, while the blue color (ϕ=0) represents the surface patches with almost no BAR domains. Grey lines on the protrusions denote the directions of the orientation of BARs. For simplicity reasons, the entropy of mixing of BAR domains was not taken into account. Shapes were calculated within the model introduced in Section 3.2 for the following values of model parameters: $C_{0}=0$, v=0.70, $C_{p}=3.0$, $K_{p}L_{0}/2=k_{c}$.

The external force on the membrane is modelled as a rod-like structure, which grows inside the cell/vesicle and stretches it as schematically shown in Figure 23. In numerical simulations, this is achieved by setting the constraint on the minimal height of the closed membrane shape. Such external force could be applied experimentally, for example by the tip of the cantilever to which the force of the atomic force microscope is applied [41], or it could be a consequence of growing/elongating actin cytoskeleton inside the vesicle or cell [18,124,183]. Note that when the cell/vesicle is stretched by the rod-like structure, the bending energy of the membrane is increased. The limitation of our simulations is that we did not consider the competition between the membrane bending and the bending of actin filaments or actin filament bundles [184,185,186], i.e., the lenght of the actin cytoskeleton is fixed by the constraint (see Figure 23, Figure 24 and Figure 25). A large increase of the membrane bending energy as a consequence of the membrane stretching could have an effect on the length and the shape of the actin cytoskeleton structure, e.g., it could cause the buckling effect, which cannot be taken into account in our modelling (Figure 23, Figure 24 and Figure 25) [185,186,187].

The effect of mechanical force on a two-component membrane is studied in Figure 23. In this case, the membrane contains two types of nanodomains, i.e., flat nanodomains and relatively highly curved isotropic nanodomains. Equilibrium membrane shapes are calculated within the model introduced in Section 2.7 for a fixed value of the reduced volume *v*. Without the application of actin force, the equilibrium membrane shape is composed of a spherical bottom with an undulated (necklace-like) membrane protrusion (Figure 23a). The two types of membrane nanodomains are not completely laterally separated. Nevertheless, the local relative area density of highly curved isotropic nanodomains is higher in the undulated part of the membrane, which makes sense because the necklace-like part of the membrane (two smaller spheres) is actually formed as a consequence of the presence of highly curved isotropic nanodomains (Figure 23a). When an external mechanical force is applied, i.e., the membrane is vertically stretched, the protrusion gradually transforms from an undulated to tubular shape (Figure 23). The shape in Figure 23f represents almost a limit shape, composed of a spherical bottom and a tubular protrusion. Geometry dictates that tubular protrusion has to get thinner and longer if the closed membrane shape is stretched and the reduced volume *v* remains constant [23]. Note also that the degree of lateral phase separation of the two types of nanodomains is getting higher as the shape is stretched. Without the external mechanical force, the isotropic membrane nanodomains can induce/stabilize only undulated (necklace-like) protrusions (Figure 23a) [23]. Only when undulated membrane protrusions are stretched by the mechanical force of the cytoskeleton, they are converted into tubular protrusions (Figure 23f). In the absence of external force, tubular membrane protrusions can be stabilized only by anisotropic curved membrane nanodomains [23].

### 3.2. Orientational Ordering of Membrane Attached Bar Domains and the Force of Cytoskeleton

Next, we shall study the combined effect of orientational ordering of membrane attached BAR protein domains and cytoskeleton force on membrane shapes (see Figure 3 and Figure 24). The bending energy density (Equation (50)) of a flexible anisotropic banana-like BAR protein attached to the membrane can be expressed as [18]

(50) $$w_{bar}=\frac{K_{p}L_{0}}{2}{\left(C-C_{p}\right)}^{2},$$

where $K_{p}$ is the flexural rigidity and $L_{0}$ the length of the BAR domain. Curvature preference of the BAR domain is determined by its intrinsic (spontaneous) curvature $C_{p}$ [18]. This energy term was originally introduced in [86,188]. We assume that the protein has circular (radial) intrinsic shape [18].

As it can be seen in Equation (50), the energy of a rod-like BAR domain depends on its orientation ω. The orientation angle ω dependence is included in the local membrane curvature *C*, which is “seen” by the attached BAR domain. It can be expressed by Euler’s relation (Equation (51)) as:

(51) C=H+Dcos(2ω).

Note that in Equation (6), ω represents the angle between the principal systems of the tensors $\underline{C}$ and ${\underline{C}}_{m}$, while in Equation (51), ω stands for the angle of the normal plane in which the BAR domain is lying relative to the normal plane of the first principal curvature $C_{1}$ (see also Figure 24) [18]. Minimizing the bending energy of BAR domains (Equation (50)) with respect to ω, taking into account Equation (51), yields the angle corresponding to minimal $w_{bar}$ at given *H* and *D* (Equation (52)) [18]:

(52) $$\cos\left(2\omega \right)=\frac{C_{p}-H}{D}.$$

The total free energy of rod-like BAR domains (Equation (53)) depends on their local relative area density (concentration) ϕ [18]:

(53) $$F_{bar}=\int_{S}\phi w_{bar}\;dS,$$

where dS is an infinitesimal area element and the integration is performed over the entire membrane surface area *S*. To model a lipid bilayer membrane covered with certain concentration of anisotropic rod-like BAR domains, we use a Helfrich isotropic bending energy density (Equation (19)) with the addition of the above described bending energy of flexible anisotropic banana-like BAR domains (Equation (53)). Even though the membrane contains only one type of domains, i.e., BAR domains, these domains can still have different concentrations profiles on the membrane surface. Therefore, we should take into account also the entropy of mixing of BAR domains. The total energy of the system (Equation (54)) can then be written as:

(54) $$F=F_{iso}+F_{bar}+F_{mix}.$$

Note that in this case, in $F_{mix}$ (Equation (48)) ϕ stands for the local relative area density (concentration) of BAR domains. At each point of the membrane surface, ϕ is determined in the process of variation of the system’s free energy [18]. The rod-like proteins may represent BAR, F-BAR or I-BAR domains, each of them with a different intrinsic curvature. Note that BAR and F-BAR domains usually attach to the outer membrane surfaces, while I-BAR domains bind to the inner surfaces of biological membranes [18,115] (see also Figure 22).

The influence of cytoskeleton force on the closed membrane shape, applied to the membrane with the attached rod-like BAR domains, is presented in Figure 25. The equilibrium shapes were determined by minimization of the free energy *F* (Equation (54)) for a fixed value of the reduced volume *v*. We model a closed membrane shape with zero spontaneous curvature ($C_{0}=0$), which contains a fixed average relative area density (concentration) of curved BAR domains. Without the application of force, BAR domains form a relatively wide protrusion on the top (Figure 25a). The radius of the protrusion is correlated to the intrinsic curvature of BAR domains $C_{p}$ (see Equation (50)), i.e., BAR domains with higher curvature $C_{p}$ prefer to be located on thinner tubular protrusions. If the geometry allows, BAR domains usually form these protrusion in order to fit into them [18]. BAR domains in our case have a relatively low intrinsic curvature ($C_{p}=3.0$), therefore, the protrusion in Figure 25a is quite wide and not very prominent. The directions of BAR domains are schematically shown in Figure 25 as grey lines. Note that without the application of external force, BAR domains are always oriented perpendicular to the protrusion (Figure 25a) [18]. This theoretical prediction was recently confirmed in [132], where BAR domain induced tubular protrusions were observed with BARs oriented perpendicular to the axis of the protrusion. Note that in molecular dynamics simulations presented in [132], the direct interactions between BAR domains were also taken into account.

When the force of actin filaments is applied, i.e., the membrane is vertically stretched, the tubular protrusion has to get thinner and longer if the reduced volume is fixed (Figure 25) [18,23]. Thinner tubular protrusion is in this case induced by the external force of actin filaments and not by BAR domains. In order to fit into the membrane surface, BAR domains now adjust their angle of orientation. The angle of orientation of BAR domains ω is adjusted according to Equation (52). The thinner the tubular protrusion gets, more along the vertical axis are the BAR domains oriented (Figure 25). The orientation of BAR domains on tubular protrusions is therefore changed when the external mechanical force is applied to the membrane [18]. The membrane attached BAR domains oriented at a certain angle ω≠0 may form a chiral surface structure (see for example Figure 25e). Chirality plays an important role in many branches of science, for example in the development of thin anisotropic nano strips that may be transformed into nanotubes [189], or in the formation of nanotubes, which may be driven by the self-assembly of chiral amphiphiles [190,191].

### 3.3. Active Protrusive Force

Many cellular processes display that curved proteins, or curved membrane nanodomains (Figure 6 and Figure 22), are able to use the cytoskeleton of the cell in a way to give rise to additional protrusive forces, for example due to the polymerization of actin (Figure 22b) but also from other mechanisms like ion pumps [19,192,193,194]. Such curved membrane proteins with a convex shape can induce outwards bending of the membrane (Figure 22). When this budding recruits additional cytoskeletal forces which push the membrane outwards even further, membrane protrusions can be efficiently initiated. This coupling of convex spontaneous curvature and actin polymerization is emerging as an efficient cellular mechanism for the production of protrusions which are actin-based. Certain viruses are known to exploit this mechanism during their budding from the infected cell [195,196].

**Figure 26:**
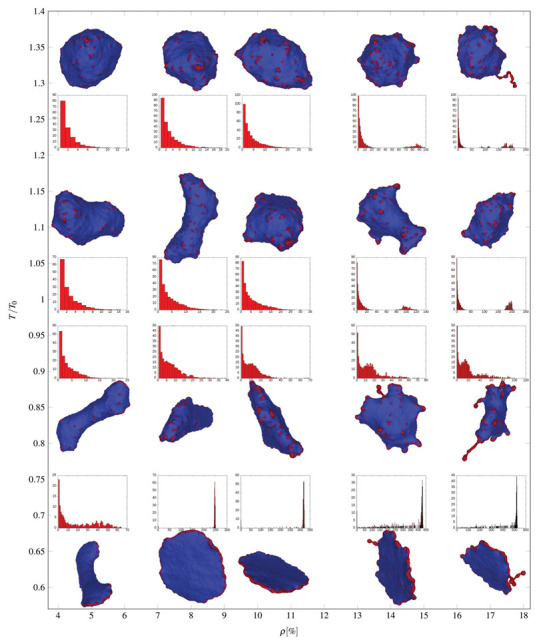
MC predicted vesicle shapes in thermal equilibrium with curved nanodomains and active protrusive force that is perpendicular to the surface and points outward at the positions of curved nanodomain clusters (red surface) with intrinsic curvature $c_{0}$. The blue surface represents the nanodomain-free lipid bilayer and has zero intrinsic (spontaneous) curvature. In the corresponding cluster-size distributions, the *y*-axis represents the ensemble averaged number of nanodomain clusters of each size, while the *x*-axis is the size of the cluster of inclusions. Adapted with permission from ref. [24]. 2019 Royal Society of Chemistry.

Due to an active force parameter *F* the additional energy term (see Equation (62) is being calculated to achieve new steady states. The results are presented in Figure 26. The protrusive force is acting only on the areas with curved nanodomains (proteins), perpendicular to the inner surface where $F=kT_{0}/l_{min}$. In comparison to Figure 17, the phase diagram is now strikingly different. Depending on the clustering of curved nanodomains, the protrusive force elongates the phospholipid vesicle for lower concentrations of proteins in the membrane. The active protrusive forces promote separation and budding of the convex curved nanodomains. Furthermore, an additional effect of the active forces is seen in Figure 26 at low temperatures, where a second transition is found below the budding transition leading to a new class of shapes that was not observed in the absence of protrusive forces (see Figure 17). The vesicles change from deformed quasi-spherical to pancake-like, flattened shapes, where all or nearly all the nanodomains aggregate at the rim, forming one large cluster resembling a ring. Such organization of nanodomains into a circular rim around a flat vesicle is highly effective in stretching out the flat parts of the vesicles (Figure 26). It was shown that flat regions are almost devoid of nanodomains, since these regions are energetically inconvenient for the curved nanodomains [24]. The stretching of the membrane in these regions also suppresses aggregation of the curved nanodomains, resulting in a high stability of the rim aggregate. Necklace-like structure occurs at higher nanodomains concentration. In constrast with the cases without protrusive forces (see Figure 17), the beads are formed also at higher membrane stiffness when there is an additional protrusive force acting on the curved nanodomains (Figure 26).

**Figure 27:**
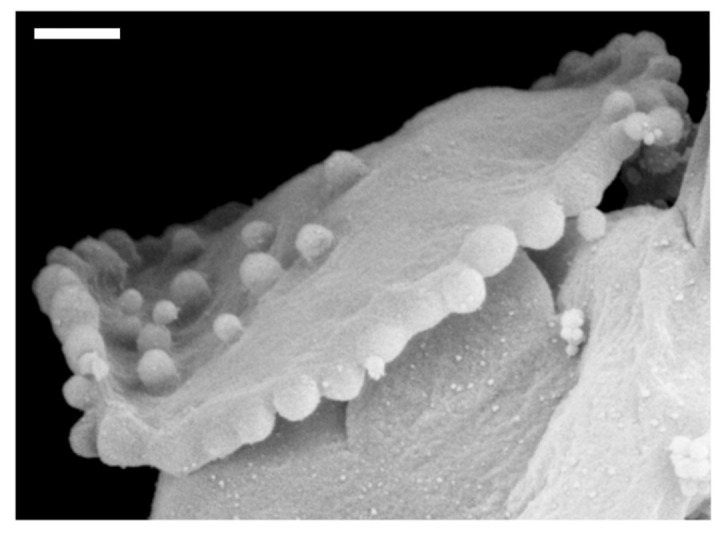
Scanning electron microscope image of a vesicular structure found in the isolate from peripheral blood of a healthy human donor. Fresh blood taken into a vacutube with anticoagulant trisodium citrate was centrifuged at 1500×*g* to sediment erythrocytes. The upper plasma, poor with platelets was then repeatedly centrifuged at high speed (17,570×*g*) and washed with phosphate and citrate buffered saline to isolate fragments of blood cells. Some residual cells, mostly platelets are usually also present in the isolate. The size and the shape of the structure indicates that it derives from a residual platelet. It is however likely that the assembly would integrate parts of membranes of other cells, e.g., erythrocytes and leukocytes during the processing of blood. Similar structures (with budding edge) of different sizes are often found in microvesicle isolates from blood. (Top bar equals 500 nm).

To conclude, flattened pancake-like shapes thus depend on the curved nanodomain concentration and membrane stiffness. By varying the model parameters we can predict similar vesicle shapes as they are found in nature. Figure 27 shows a scanning microscope image of a vesicular structure from a blood isolate. The flattened vesicle budding at the rim of the disc shape may be caused by the accumulation of curved nanodomains as predicted in Figure 26. If the nanodomain density is increased even further, budding can occur on the flat sides of the disc due to embedded protein nanodomains (Figure 26 and Figure 27). Two mechanisms may thus drive the budding process, the non-homogenous lateral distribution of nanodomains with non-zero intrinsic curvature (see Figure 14b,c) and the active protrusive force (Figure 26) [12,15,16,24,25,32,40].

Inverting the active force in MC simulations to act into the membrane interior with nanodomains with negative intrinsic curvature produces interesting results (see Figure 14). Without the active force (Figure 14a) we predicted the inverted budding into endovesicles connected to the parent membrane by a thin neck. However, when the active force is added, a large invagination is predicted (see Figure 14b,c). Note that the neck area in Figure 14a is free of curved nanodomains. The membrane curvature in the neck region is anisotropic and therefore not favorable for highly curved isotropic nanodomains/inclusions. For long term stable shapes that feature a connective neck between the bud and the parent membrane, we would have to take into account the role of anisotropic saddle-like nanodomains, which would assemble in the neck region [15,81,89,168,169] as shown in Figure 21 [170].

## 4. Discussion and Conclusions

We described the impact of different membrane curved nanodomains and passive and active forces of cytoskeleton and membrane skeleton on closed membrane shapes and membrane budding. Our main interest was the role of passive and active forces in the formation of tubular protrusions on the membranes of cells/vesicles. Such forces may be a consequence of elongating actin cytoskeleton inside the closed membrane [18,71,124]. However, it was shown that membrane tubular protrusions can be induced also in the absence of force if the membrane contains anisotropic curved nanodomains [23]. Note that even in that case, actin filaments may fill the space inside the tubular protrusion and promote a long term stabilization of the protrusion (Figure 22) [71,93].

First, we studied the effect of different types of curved nanodomains on closed membrane shapes in the absence of cytoskeleton force. We presented how anisotropic cylinder-like nanodomains may enforce the formation of a tubular protrusion in Figure 19. In Figure 20, we demonstrated the formation of an isotropic bud as a consequence of highly curved isotropic nanodomains. We presented also the effect of anisotropic saddle-like nanodomains, which facilitate the formation of anisotropic neck connecting two parts of the vesicle (Figure 21). In this case, anisotropic saddle-like nanodomains assemble in the neck region and enhance the long term stabilization of the neck connecting the bud and the parent membrane [15,81,89,168,169].

Next, we demonstrated the effect of actin filaments force in the case of a two component membrane with flat and highly curved isotropic protein induced nanodomains (Figure 6). Without the force of actin filaments, the membrane is composed of a spherical bottom and an undulated (necklace-like) protrusion (Figure 18 and Figure 23) induced by highly curved isotropic membrane nanodomains. Isotropic nanodomains can induce/stabilize only undulated membrane protrusions but not membrane tubular protrusions [23]. If an undulated membrane protrusion is stretched by a mechanical force, it is transformed into a tubular protrusion (Figure 23). In this process, the lateral separation of the different types of membrane components and nanodomains becomes more pronounced. When the cell/vesicle membrane is stretched, tubular protrusion becomes thinner and longer (Figure 23). The actin force can in this way induce the growth of membrane tubular protrusions on closed membrane shapes (Figure 23) [23].

Furthermore, the effect of cytoskeleton force on a membrane with anisotropic rod-like BAR domains is presented in Figure 3. Due to their anisotropic properties, BAR domains are able to induce membrane tubular protrusions even in the absence of cytoskeleton force (Figure 25a). Actin cytoskeleton stretching force can induce even more pronounced (longer and thinner) tubular protrusions, leading to the change of the average orientation angle of BAR domains on the protrusion (Figure 25) [18]. Note that when the membrane is stretched, the cylindrical membrane protrusion has to become longer and thinner in order to keep the constant value of the reduced volume of the cell/vesicle. When that happens, the principal curvature of the membrane tubular protrusion $C_{2}=1/R_{p}$ ($R_{p}$ is the radius of the protrusion) becomes larger than the intrinsic curvature of BAR domains $C_{p}$. In order to fit to the membrane and lower the energy, BAR domains now adjust their angle of orientation (Figure 25) (see also Equation (52)). Note that without the force, BAR domains would never induce a protrusion with larger curvature than their own intrinsic curvature $C_{p}$ because that would not be energetically favorable. Therefore, only mechanical force is able to induce the change of orientation angle of BARs [18].

It is still not fully understood how the membrane protrusions are actually induced or stabilised by a mechanical force of actin filaments [18,93]. In this review described mechanisms of membrane protrusion growth could be tested experimentally by measuring the average orientation of BAR domains on the membrane tubular protrusions. If the average orientation of the membrane attached BAR domains would be different from ω=0 (see Figure 24) in the absence of the experimentally induced external force, that would mean that the role of actin filaments is not only to stabilize, but also to physically stretch the membrane and induce the process of the protrusion growth [18].

We have also explained how the stability of the echinocyte shape of RBCs is modulated by competition between the membrane Helfrich local bending energy and the shear energy of the membrane skeleton [44,45]. We have shown that the spiculated RBC shape (Figure 12) can be determined by minimization of the sum of shear and bending energies. Discocyte and echinocyte shape transformation of RBCs are predicted to be driven by changing the optimal area difference between the lipid monolayers [26]. The optimal area difference is determined through the differences in the area per molecule, the difference in the number of molecules and the difference in the intrinsic molecular shapes in the inner and the outer monolayer [28,164].

We further presented the stability analysis of competition between prolate and oblate (discocyte) RBC shapes. The existing theoretical models [46,60,72,73,140,154] fail to explain a relatively broad range of reduced volume values for which discocyte red blood cells are experimentally observed [150]. We demonstrated that by taking into account the in-plane orientational ordering of membrane components with direct interactions and the extrinsic (deviatoric) curvature elasticity, the volume range of stable discocyte RBC shapes could be significantly increased [137] (Figure 11). Nematic-like orientational ordering in RBC membrane could be present for example due to lipid anisotropy (Figure 1 and Figure 4) and/or due to protein induced anisotropic membrane nanodomains (Figure 6, Figure 7 and Figure 10) [12]. Wide stability range of calculated discocyte RBC shapes (Figure 11) due to extrinsic (deviatoric) term in the free energy expression is a consequence of their unique shape. In the equatorial region of discocytes, the difference between the two principal curvatures is large so the extrinsic (deviatoric) term enforces strong orientational order in that region, which contributes to the lower total free energy of discocyte shape [137].

We showed that vesicle spherical or necklace protrusion growth can be predicted within a simple analytic theory of self-assembly of isotropic curved membrane nanodomains [26,84]. If isotropic curved membrane nanodomains are assumed to be laterally mobile throughout the membrane, we showed that above a critical concentration of nanodomains protrusion growth is energetically favorable. At low densities of curved membrane nanodomains and low temperature, the vesicle equilibrium shapes remain quasi-spherical. Higher densities of curved membrane nanodomains result in an increase of cluster sizes, promoting budding and protrusion growth on the membrane. An example of such a budding promotion can be seen in Figure 20. With increasing fraction of membrane constituents that favor strong curvature, a budding of the smaller sphere is promoted with a notable redistribution of components.The theory of self-assembly is also in good agreement with Monte-Carlo (MC) simulations of three-dimensional equilibrium closed shapes of vesicles (Figure 17).

Additionally, we have presented the results of vesicle steady-states with active force that acts at the locations of curved nanodomains (Figure 22). When the curved nanodomains are isolated or accumulated in very small clusters, the membrane is relatively flat and the active protrusive force stimulates the aggregation of small clusters of curved nanodomains, since the active force points at the outwards normal at each curved nanodomain, thereby promoting the outwards deformation induced by the curved nanodomain. However, for larger clusters of highly curved nanodomains, the active forces point in various directions, which inflates and deforms the cluster. The presence of the active protrusive forces may give rise to the formation of aggregates of curved nanodomains and to budding at higher temperatures and lower average curved nanodomain densities, compared to the passive system in thermal equilibrium (no active forces). The more robust aggregation and budding as a result of the recruited cytoskeleton active forces may have important consequences for different biological processes, like budding of viruses and initiation of cellular protrusions during development and cell motility.

## 5. Materials and Methods

### 5.1. Calculation of Axisymmetric Closed Membrane Shapes

**Figure 28:**
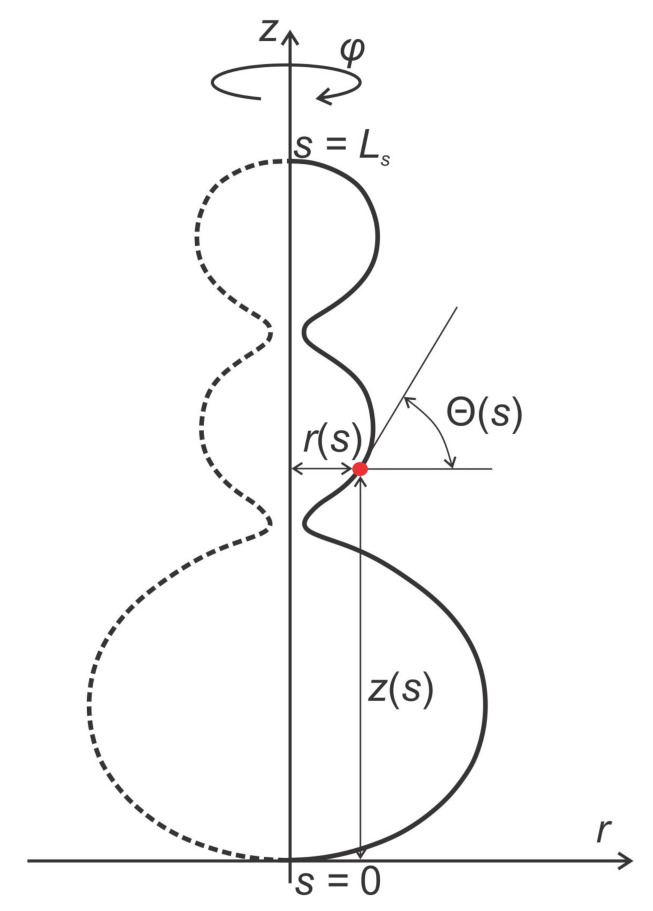
The shape cross-section in r−z plane. The shape profile radius r(s) and the height z(s) at the given arc length *s* are calculated from Θ(s) via Equations (55) and (56). $L_{s}$ is the length of the shape profile and φ is the angle of rotation around the *z*-axis.

In this Subsection we discuss the method used to calculate equilibrium axisymmetric closed membrane shapes presented in Figure 11, Figure 19, Figure 20 and Figure 21, Figure 23 and Figure 25. These shapes are assumed to have the rotational symmetry about the vertical *z*-axis. In order to represent the surface of axisymmetric closed membrane shapes, we need to define a profile curve in the r−z plane (Figure 28). The surface of the cell/vesicle is constructed by rotating that profile curve about the *z*-axis by the angle φ=2π. The profile curve is parameterized with the angle Θ(s) of the line tangent to the profile curve relative to the plane that is perpendicular to the axis of rotation *z*. Here, *s* stands for the arc length of the profile curve [197] (Figure 28). For a given function Θ(s), the shape profile radius r(s) (Equation (55)) and the height z(s) (Equation (56)) are calculated according to [18,23]:

(55) $$r\left(s\right)=\int_{0}^{s}\cos\left(\Theta \left(s^{\prime }\right)\right)\;ds^{\prime },$$

(56) $$z\left(s\right)=\int_{0}^{s}\sin\left(\Theta \left(s^{\prime }\right)\right)\;ds^{\prime }.$$

To describe the shape contour Θ(s) (see Figure 28) we use a function approximated by the Fourier series (Equation (57)) [198],

(57) $$\Theta \left(s\right)=\Theta_{0}\frac{s}{L_{s}}+\sum_{i=1}^{N}a_{i}\sin\left(\frac{\pi }{L_{s}}i\cdot s\right),$$

where $L_{s}$ is the length of the shape profile (Figure 28), *N* is the number of Fourier modes and $a_{i}$ are the Fourier amplitudes. To ensure that the axisymmetric membrane shape is closed and smooth, the following boundary conditions are applied: Θ(0)=0, $\Theta (L_{s})=\pi$, $r\left(0\right)=r\left(L_{s}\right)=0$. In Equation (57), $\Theta_{0}$ is the angle at the north pole of the vesicle (Figure 28), $\Theta_{0}=\Theta \left(L_{s}\right)=\pi$ [18,23]. In analogy to the area density profile for laterally separated mixtures, we assume that the local relative area density of the nanodomains *A* (Figure 19, Figure 20 and Figure 21 and Figure 23) or BAR domains (Figure 25) has the form (Equation (58)) [89,199,200]:

(58) $$\phi \left(s\right)=\left(\phi_{2}^{A}-\phi_{1}^{A}\right)\left[-\tanh\left(\chi (s-s_{0})\right)+1\right]/2+\phi_{1}^{A},$$

where the cell/vesicle surface is divided into two distinct regions, characterized by the minimal and the maximal local area densities of the nanodomains *A* or BAR domains, $\phi_{1}^{A}$ and $\phi_{2}^{A}$, respectively [18,23]. The parameters χ and $s_{0}$ determine the width and the position of the border between those two regions [89,199,200]. In calculations presented in Figure 19, Figure 20 and Figure 21, Figure 23 and Figure 25, we used a constraint on average relative area density (concentration) of the components *A* (Figure 19, Figure 20 and Figure 21 and Figure 23) and BARs (Figure 25). The average relative area density $\phi_{ave}$ (Equation (59)) is calculated as the following integral over the whole membrane surface:

(59) $$\phi_{ave}=\int_{0}^{2\pi }d\varphi \int_{0}^{L_{s}}\phi \left(s\right)r\left(s\right)ds\;/S,$$

where *S* is the surface area of the cell/vesicle [18,23]. Note that in the case with membrane attached BAR domains, the remaining surface area of the vesicle (not covered with BARs) is not covered by anything, while in the case of a two-component membrane, the remaining surface area (not covered by the component *A*) is fully covered by the component *B*.

The main focus of this paper is the impact of external actin cytoskeleton force on cell/vesicle shapes. Actin cytoskeleton is modelled as a rod-like structure, which stretches the membrane from the inside (see Figure 23 and Figure 25). To study the shapes of cells/vesicles elongated by an external force, we add a constraint of minimal vertical distance between the poles of the shape [18,23,197,200].

By taking into account the Equations (57) and (58), the minimisation of the free energy functional (Equations (49) and (54)) is replaced by the minimisation of function with many variables. In our case, the variables are the Fourier amplitudes $a_{i}$, the shape profile length $L_{s}$, and the parameters χ, $s_{0}$, $\phi_{2}^{A}$, $\phi_{1}^{A}$. The key parameters involved in most of the membrane free energy functionals, the principal curvatures $C_{1}$ and $C_{2}$, for axisymmetric shapes, are given as $\frac{d\Theta (s)}{ds}$ and $\frac{\sin(\Theta (s))}{r(s)}$, respectively [18,23].

The equilibrium closed membrane shapes are obtained by numerical minimisation of the membrane free energy functional *F* (see Equations (49) and (54)) at non linear constraints for the reduced volume *v*, the minimal vertical distance between the poles of the shape, and the average relative area density of membrane components (either the nanodomains *A* or the BAR domains) $\phi_{ave}$ [18,23]. Functions Θ(s) and ϕ(s) are obtained as a result of the described minimisation procedure. Function Θ(s) describes the shape of the cell/vesicle, while function ϕ(s) describes the relative area density (concentration) distribution of the membrane components on the membrane surface (BARs, lipids or nanodomains *A* and *B*) [18,23].

### 5.2. Monte-Carlo Simulations of Closed Membrane Shapes

As an example of another approach to the modelling of closed membrane shapes, we introduced the triangulated mesh model for the membrane [201]. Simulations using a Metropolis-Hastings Monte-Carlo method [12,24,201,202] may serve as a basic approach for determination of vesicle shape development. The model for the discretization of a closed vesicle surface is a triangulated mesh, consisting of vertices, connected with N bonds of length between $d_{\min}$ and $d_{\max}=1.7\;d_{\min}$, forming triangles on the surface. The bilayer membrane can be treated in the first approximation as a two dimensional liquid layer [203].

The closed vesicle shape is developed into a thermal equilibrium state. The shape evolution is measured in Monte-Carlo sweeps (MCSs). One MCS consists of an individual move of each of the *N* vertices by a random displacement in the sphere with a radius $s=0.15\;d_{\min}$—we will refer to this action as the vertex move. To preserve membrane “fluidity”, bond flipping is maintained within a triangulated network. In each MCS, the moving of the vertex attempt is followed by 3N attempts to flip a bond chosen at randomly. A single bond flip includes four vertices of two neighboring triangles. The tether between two vertices is cut and then reestablished between the other two vertices which were initially unconnected (for details see [202]). Each individual Monte-Carlo step (either vertex move or bond flip) is accepted with a probability $\min\left[1,\exp\left(-\Delta W/kT\right)\right]$ according to Metropolis-Hastings algorithm, where ΔW is the change of energy due to the vertex move or bond flip. The main parameter of the model that defines mechanical bilayer properties is the bending rigidity constant.

The energy is a sum of three components (Equation (60)):

(60) $$W=W_{b}+W_{d}+W_{F},$$

where $W_{b}$ is the local bending energy of the membrane, the energy of the direct interaction between membrane nanodomains with the given intrinsic curvature (vertices) is $W_{d}$ and $W_{F}$ is the energy due to active force acting on the membrane.

The standard Helfrich expression is used for the bending energy $W_{b}$ of the membrane [73] and rewritten for a tensionless membrane (Equation (15)) in integral form with mean curvature expressed as individual principal curvatures. The membrane keeps fixed topology, so the Gaussian curvature contribution to the change of bending energy is left out from the expression.

In the model the attraction force between the neighboring membrane nanodomains/ inclusions (vertices) with intrinsic curvature $C_{0}$ contribute to an additional energy term (Equation (61)) [24]:

(61) $$W_{d}=-w\sum_{i<j}H\left(r_{0}-r_{ij}\right),$$

where *w* marks a direct interaction constant between two neighboring nanodomains and is directly proportional to the strength of interaction. The energy is summed over all nanodomain pairs where $r_{ij}$ is their in-plane distance, and H(r) is a Heaviside step function. The range of direct interaction is given by $r_{0}$.

The energy contribution of the local protrusive active forces (Equation (62)) can be decribed as [24]:

(62) $$W_{F}=-F\sum_{i}{\hat{n}}_{i}\cdot x_{i}$$

where the magnitude of the force is *F*, ${\hat{n}}_{i}$ is the normal facing outwards to the membrane at the location of the *i*-th vertex (*i*-th nanodomain) and $x_{i}$ is the position vector of the *i*-th vertex (inclusion). The sum runs over all nanodomains/inclusions in the membrane.

## Abbreviations

The following abbreviations are used in this manuscript:

ATP	Adenosine triphosphate
RBC	Red blood cell
NMIIA	Non-muscle myosin IIA
DE	Deviatoric elasticity
BAR	Bin/Amphiphysin/Rvs
MC	Monte-Carlo
